# Mutations in *SPATA13/ASEF2* cause primary angle closure glaucoma

**DOI:** 10.1371/journal.pgen.1008721

**Published:** 2020-04-27

**Authors:** Naushin H. Waseem, Sancy Low, Amna Z. Shah, Deepa Avisetti, Pia Ostergaard, Michael Simpson, Katarzyna A. Niemiec, Belen Martin-Martin, Hebah Aldehlawi, Saima Usman, Pak Sang Lee, Anthony P. Khawaja, Jonathan B. Ruddle, Ameet Shah, Ege Sackey, Alexander Day, Yuzhen Jiang, Geoff Swinfield, Ananth Viswanathan, Giovanna Alfano, Christina Chakarova, Heather J. Cordell, David F. Garway-Heath, Peng T. Khaw, Shomi S. Bhattacharya, Ahmad Waseem, Paul J. Foster

**Affiliations:** 1 NIHR Biomedical Research Centre at Moorfields Eye Hospital and UCL Institute of Ophthalmology, London, United Kingdom; 2 Moorfields Eye Hospital NHS Foundation Trust, City Road, London, United Kingdom; 3 UCL Institute of Ophthalmology, Bath Street, London, United Kingdom; 4 Department of Ophthalmology, St. Thomas’ Hospital, Westminster Bridge Road, London, United Kingdom; 5 Centre for Oral Immunobiology and Regenerative Medicine, Institute of Dentistry, Queen Mary University of London, London, United Kingdom; 6 Medical Genetics Unit, St. George’s University of London, Cranmer Terrace, London, United Kingdom; 7 Genetics and Molecular Medicine, King’s College London, Great Maze Pond, London, United Kingdom; 8 Blizard Advanced Light Microscopy, Blizard Institute, Queen Mary University of London, London, United Kingdom; 9 Department of Ophthalmology, University of Melbourne, Victoria, Australia; 10 Department of Ophthalmology, Royal Free Hospital NHS Foundation Trust, Pond Street, London, United Kingdom; 11 Society of Genealogists, Goswell Road, London, United Kingdom; 12 Institute of Genetic Medicine, Newcastle University, Newcastle Upon Tyne, United Kingdom; University of California, San Francisco, UNITED STATES

## Abstract

Current estimates suggest 50% of glaucoma blindness worldwide is caused by primary angle-closure glaucoma (PACG) but the causative gene is not known. We used genetic linkage and whole genome sequencing to identify Spermatogenesis Associated Protein 13, *SPATA13* (NM_001166271; NP_001159743, *SPATA13* isoform I), also known as *ASEF2* (Adenomatous polyposis coli-stimulated guanine nucleotide exchange factor 2), as the causal gene for PACG in a large seven-generation white British family showing variable expression and incomplete penetrance. The 9 bp deletion, c.1432_1440del; p.478_480del was present in all affected individuals with angle-closure disease. We show ubiquitous expression of this transcript in cell lines derived from human tissues and in iris, retina, retinal pigment and ciliary epithelia, cornea and lens. We also identified eight additional mutations in S*PATA13* in a cohort of 189 unrelated PACS/PAC/PACG samples. This gene encodes a 1277 residue protein which localises to the nucleus with partial co-localisation with nuclear speckles. In cells undergoing mitosis SPATA13 isoform I becomes part of the kinetochore complex co-localising with two kinetochore markers, polo like kinase 1 (PLK-1) and centrosome-associated protein E (CENP-E). The 9 bp deletion reported in this study increases the RAC1-dependent guanine nucleotide exchange factors (GEF) activity. The increase in GEF activity was also observed in three other variants identified in this study. Taken together, our data suggest that SPATA13 is involved in the regulation of mitosis and the mutations dysregulate GEF activity affecting homeostasis in tissues where it is highly expressed, influencing PACG pathogenesis.

## Author summary

Glaucoma is the leading cause of irreversible blindness globally. Angle closure glaucoma accounts for 50% of all glaucoma blindness impacting quality of life and burden on health services. A number of variations in DNA appear to influence the risk of the disease. However, the biological mechanism underlying this important disease remains unclear. In this paper, we report the identification and functional characterisation of the first gene, mutation in which causes primary angle closure glaucoma in a seven generation Caucasian family. We have identified other variants in the same gene in another family and individuals with the disease. This gene is involved in cell division and is highly expressed in parts of the eye affected by the disease. Mutations in this gene appear to affect important enzyme activity involved in cell division. Identification of the disease-causing role of mutations in this gene helps to further the understanding of glaucoma aetiology and identifies potential therapeutic targets for disease management.

## Introduction

Glaucoma is the most common cause of irreversible blindness worldwide, affecting nearly 80 million people [1]. It is characterised by an intermittently progressive optic neuropathy, often culminating in loss of vision if left untreated [2]. Two main subtypes are primary open angle glaucoma (POAG) [3] and primary angle closure glaucoma (PACG)[2]. PACG accounts for a quarter of all glaucoma, but causes half of all glaucoma blindness [4]. Physical obstruction of the aqueous humor outflow channels and consequent elevation of pressure inside the eye, are hallmarks of this disease. Typically PACG occurs in eyes that are smaller than average. [5].

Two PACG loci are recognized—GLC2A in a Singaporean family on 10q identified by genetic linkage analysis [6] and another locus mapped to the 3q27.1 region [7] using a population-based GWAS study in Singaporean Malay, Indian and Beijing Chinese populations. However, no causative gene has been identified for PACG at these loci. Vithana and co-workers found a genome-wide significant association between PACG and three loci: rs11024102 in pleckstrin homology domain containing family A member 7 (*PLEKHA7*) on chromosome 11p, rs3753841 in *COL11A1*on chromosome 1p, and rs1015213 located between *PCMTD1* and *ST18* on chromosome 8q in a mixture of Far East, Middle East, Indian and UK populations [8, 9]. The two single nucleotide polymorphisms (SNPs) in *PLEKHA7* and *COL11A1* were replicated in a large PACG cohort in China [10], Australia and Nepal [11]. Variants in *PLEKHA7*, an adherens junction protein, increase the risk of sudden, symptomatic pressure rises in PACG [10]. This protein is expressed in iris, ciliary body, and choroid [12] and has GTPase-activating protein (GAP) activity [13]. SNPs in other candidate genes, including *HGF* (hepatocyte growth factor) [14], *HSP70* (heat-shock protein 70) [15], *MFRP* (membrane type frizzled related protein) [16], *eNOS* (endothelial nitric oxide synthase) [15] and *MMP9* (matrix metalloproteinase-9) [17] also have significant association with PACG.

Next generation sequencing has renewed interest in using family data to identify causal gene(s) for otherwise complex disorders. It exploits information about co-segregation, thus helping to identify potentially causal variants. Rare variants of large effect influencing the susceptibility to the disease are present at a higher frequency in affected than in unaffected individuals in families, as well as the general population. Using this strategy, we have identified *SPATA13* as the causative gene for PACG in a large multigenerational Caucasian family and shown that this protein is likely to influence tissue homeostasis.

## Results

### Pedigree recruitment

**Fig 1:**
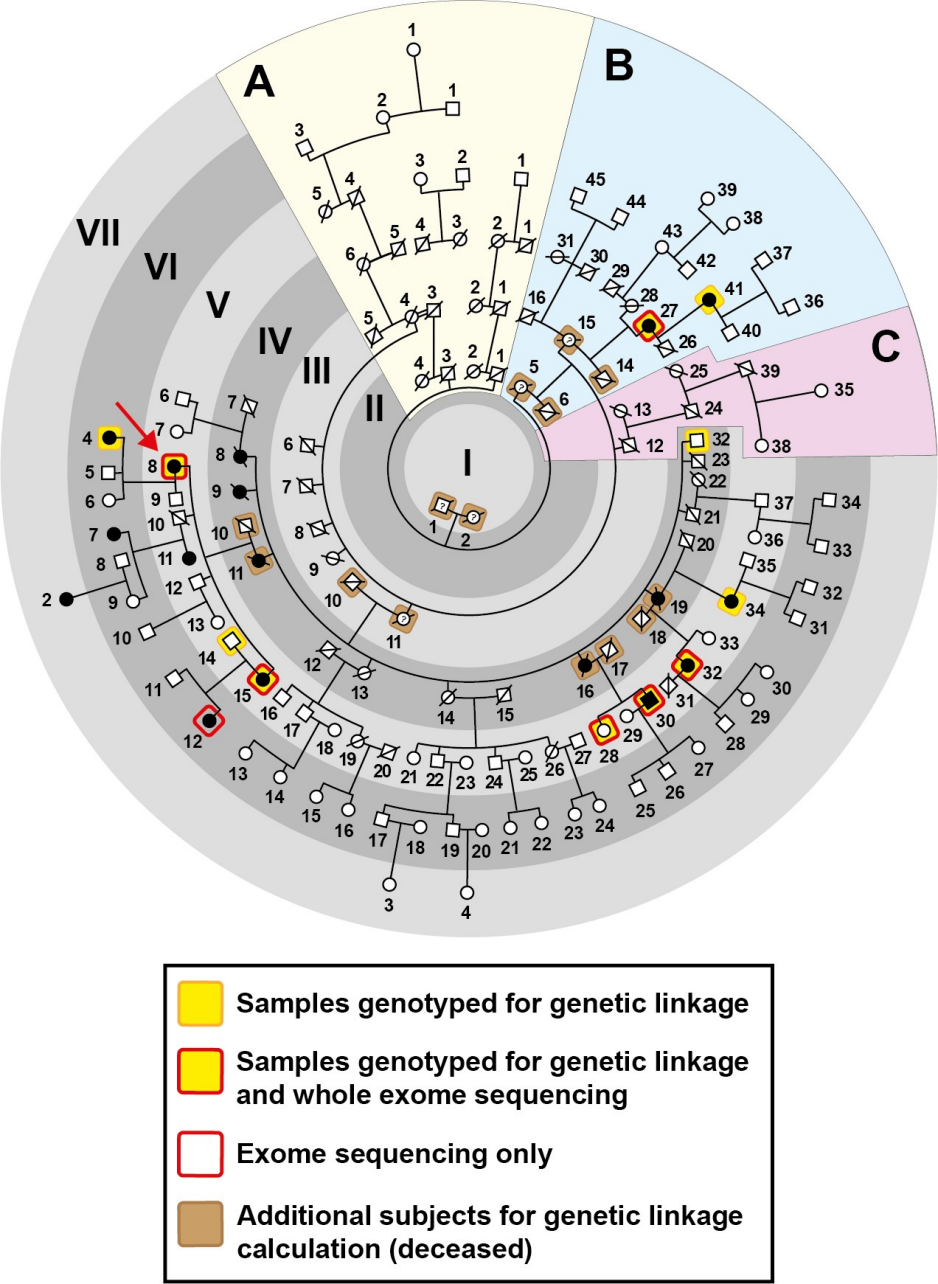
Pedigree of Family 1. The proband is V:8 (red arrow) and this pedigree shows the relationship of the 62 individuals examined (see **S1 Table** for detailed phenotype). There was no male-to-male transmission observed, and the only affected male was V:30, a first cousin of the proband. From branch B, two out of nine individuals examined were affected. There were no individuals examined from branches A and C affected with angle-closure disease. Twelve subjects, four of whom were affected first cousins, were genotyped for genetic linkage analysis, and some were analysed in greater detail by exome and whole genome sequencing. It is suspected that the phenotype in VII:2 and VI:7 is caused by another genetic defect as it is very likely that their disease is inherited from the married in spouse (V:11).

**Fig 2:**
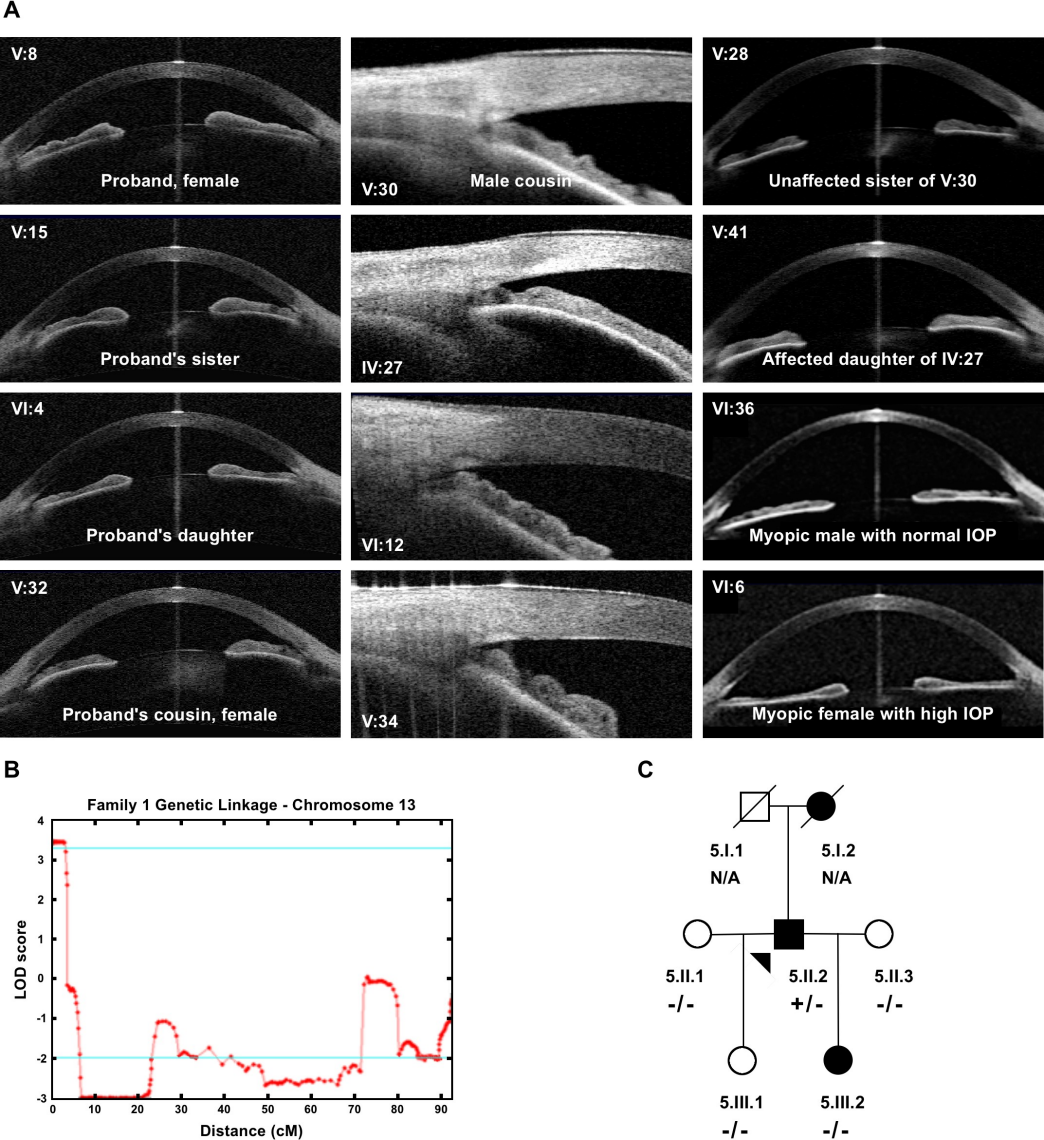
Clinical features and linkage analysis of Family 1. (A) Plateau iris configuration was observed for all nine clearly affected individuals V:8, V:15, VI:4, V:32, V:30, IV:27, VI:12 and V:34, V:41) on gonioscopy. In the first two columns, closed anterior chamber drainage angles are observed. The middle column shows high resolution AS-OCT angle imaging, where the bulky ciliary body typical of plateau iris is observed. Individual IV:27 is the oldest and most severely affected PACG individual, showing some additional iris convexity. Individuals V:28, VI:36 and VI:6 have open drainage angles and are shown for comparison. (B) Maximum multipoint LOD score of 3.5 achieved at chromosome 13 by Superlink-Online SNP-1.1. (C) Pedigree of the family 5 identified with the 9 bp deletion. The proband, 5.II.2, shown by an arrowhead carry the deletion. His mother, 5.I.2 was affected but no DNA was available for analysis. Proband has an affected daughter, 5.III.2, but her eye phenotype is different to his. Her mother, 5:II.3, has narrow but open angles and was hyperopic and it is therefore suspected that the daughter has inherited her disease from the mother.

A seven-generation Caucasian British family (Family 1) was recruited at Moorfields Eye Hospital (MEH). The proband V:8 (**Fig 1**; red arrow) attended with her sister V:15 and daughter VI:4. They gave a strong family history of glaucoma (**S1 Table**). Proband V:8 had been treated for ocular hypertension for 15 years from age 52. On examination all three, V:8, V:15 and VI:4, were found to have the same phenotype of plateau iris configuration (PIC, **Fig 2A**). The proband’s mother IV:11 underwent surgery for PACG. Including her mother, the proband had nine other aunts and uncles, i.e. ten siblings in generation IV who lived to adulthood, consisting of seven sisters and three brothers. Establishing a detailed pedigree enabled us to expand the family tree to seven generations and contact 63 members. Twelve of these (including V:11, unrelated, spouse) had occluded drainage angles (primary angle-closure suspect—PACS), PAC (Primary angle-closure disease) or PACG (see **S1 Text**). The family had lived in the East London area since at least 1812.

### Linkage analysis

All family members with occluded drainage angles or PACG were selected for linkage analysis (see **S1 and S2 Text**). An autosomal dominant disease transmission was observed in Family 1, with more affected females than males. This may be attributable due to the majority of the offspring of III:11 being female (seven daughters and three sons in adulthood; five out of seven daughters in generation IV were affected) but could also reflect the greater predisposition of women because of their smaller eyes. Individual VI:7 was not included in this analysis as her mother, V:11, not related to this family and married-in spouse of V:10, is affected. For similar reasons, her daughter (VII:2) was also not included as there could be another gene responsible for the disease, which could come through V:11. Eleven samples from this family (IV:32, IV:27, V:8, V:14, V:15,V:28, V:30, V:32, V:34, V:41, VI:4 highlighted in yellow in **Fig 1**) were genotyped on the Human CytoSNP-12 (298,199 markers) on the Illumina platform. Superlink Online SNPv1.1 was used to investigate genetic linkage in this family assuming a disease allele frequency of 1%.

An affected-only multipoint linkage analysis was performed using IV:27, V:8, V:15, V:30, V:32, V:34, V:41, VI:4 **(S2 Table).** After removal of uninformative markers, highest positive LOD score was observed on chromosome 13 between rs9580111 and rs1160226 (Max LOD Score 2.90, NPL Spair 3.80, NPL ALL 18.7). LOD scores above 1 were also observed on chromosome 5 between rs1478449—rs3812042 (Max LOD score 1.25, NPL Spair 1.44, NPL ALL-0.75), chromosome 9 between rs10979427- rs419097 (Max LOD score 1.72, NPL Spair 1.47, NPL ALL 1.76). On chromosome 13 these values increased to Max LOD score 3.5, NPL Spair 4.7, NPL ALL 34.5 when the unaffected individual IV:32, who at 94 years of age did not show any signs of PACG, was included (allowing for 90% penetrance in the parametric model) (**Fig 2B**). Including another unaffected subject V:28 also gave similar results (Max LOD score 3.760913, NPL-Spair 4.81, NPL ALL 37.12). A proximal crossover at rs10400673 and rs2166431 at the distal end in individual V:8 narrowed the genetic interval to 4.1 Mbp.

### Whole exome and genome sequencing

Four “clearly affected” members of this family, V:8, V:30, V:32 and VI:12 were analysed further with exome sequencing using SureSelect Target Enrichment System, followed by sequencing on a HiSeq2000 (Illumina) with 100bp paired end reads. The average ratio of reads alignment to reference genome was 71%. When measured at a minimum depth of 20x, 93% of the intended targets were covered. Initial filtration of variants (total number of variants in 4 samples was 98,808) was performed to identify heterozygous variants (60,329) shared between the four affected individuals (reduced to the total number to 4,836 variants, i.e. 1,209 candidate variants per sample), followed by selecting all the shared variants with MAF of <0.01. Only one variant, *SPATA13* (NM_001166271, chromosome 13q) c.1432_1440del; p.478_480del fulfilled the filtering criteria. On Sanger sequencing it was found that this variant segregated in all the nine clearly affected samples. The variant was absent in 11 of the 16 blood-related, unaffected family members and none of the spouses carried the variant. Results from five of the unaffected subjects were inconclusive due to poor quality of DNA. Two subjects with uncertain diagnosis and three subjects with other ocular conditions also carried this change. (**S1 Table**).

The 9 bp in-frame deletion in *SPATA13* results in deletion of three amino acids, QSP. There are three repeats of QSP within this region; the mutation deleted one of them. This variant had a MAF of 0.0001158 in Kavier, 0.0006118 in gnomAD Exomes, and 0.0008671 in gnomAD genomes. We also analysed V:15, V:30, IV:27 and V:28 by whole genome sequencing on HiSeqX with 30x coverage as some genes showed poor coverage on WES within the linked region on chromosome 13. The variants in the affected (V:15, V:30, IV:27) were compared with the unaffected V:28. The only variant with an allele frequency of less than 0.01 that was shared in all affected individuals within the linked regions was *SPATA13* (NM_001166271) c.1432_1440del; p.478_480del.

### Prevalence of *SPATA13* mutations in unrelated patient cohort

To identify additional variants in *SPATA13*, all 13 coding exons were screened by Sanger sequencing in a cohort of 189 unrelated patients: PACS (n = 14)/PAC (n = 106, 52 acute) and PACG (n = 69, 3 acute). One patient, (5:II:2, **Fig 2C, S3 Table**) with PACS carried the same 9 bp *SPATA13* deletion (c.1432_1440del; p.478_480del) identified in Family 1 (**S3 Table**). Genotype analysis of SNPs within *SPATA13* and its adjacent region show that this individual was not related to Family 1. The genotypes and segregation of this mutation in this family and the clinical details are given in **S3 Text**.

**Fig 3:**
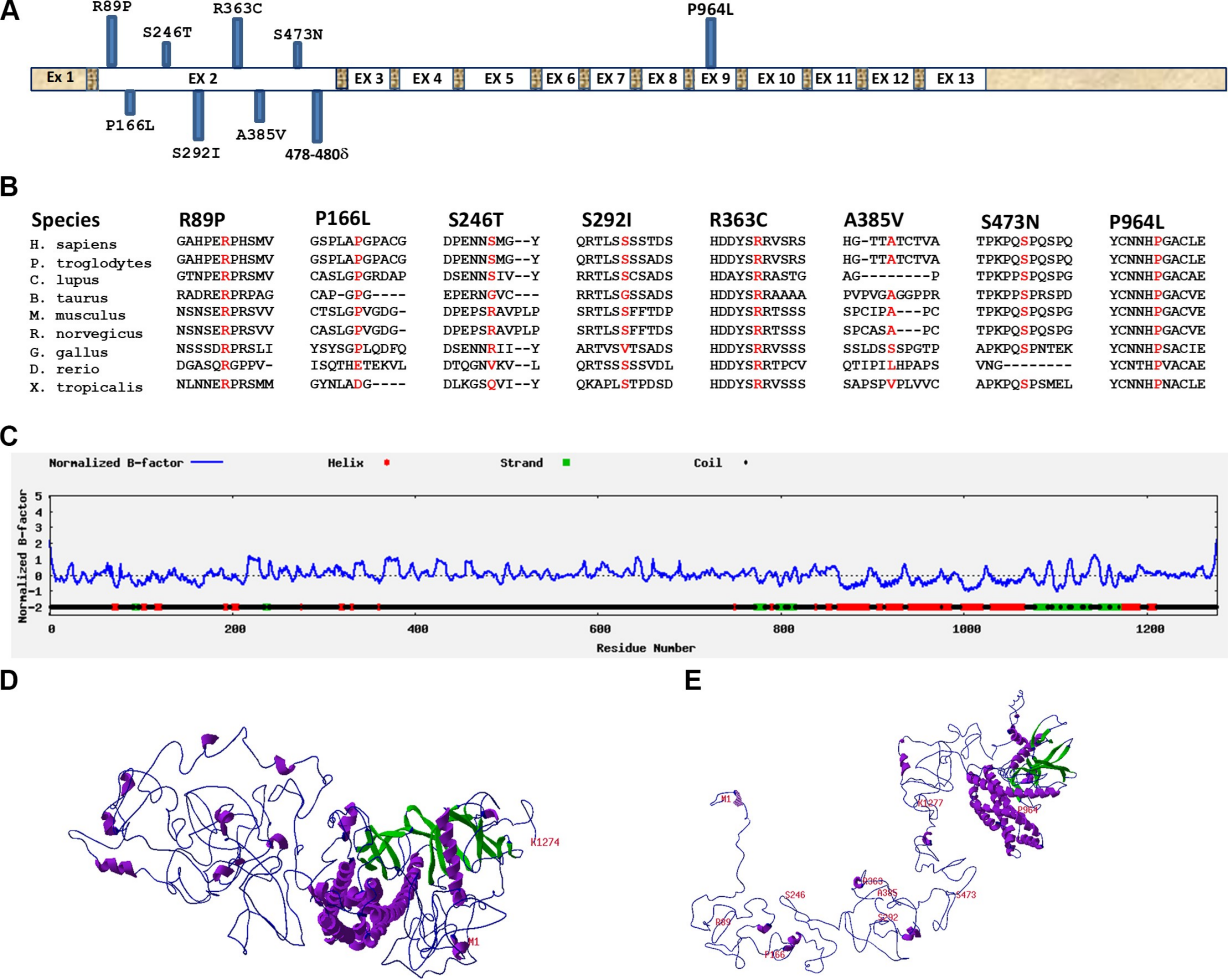
Location and predicted effect of mutations in SPATA13 gene on protein conformation. (A) Structure of SPATA13 gene structure and location of 8 mutations in exon 2 and the 9^th^ mutation in exon 9. (B) Conservation of amino acids mutated in PACG in 9 different species. (C) Secondary structure prediction for the amino acid sequence of SPATA13 polypeptide. (D) Predicted tertiary structure of SP-1277 containing the 9bp (p. 478-480del) deletion. (E) Wildtype SP-1277 showing the location of all the missense variants.

We also identified another eight variants in *SPATA13* amongst eight unrelated patients (**Table 1, Fig 3A**), seven of them were in exon 2, the largest in this gene. In our cohort the prevalence of individuals with a rare *SPATA13* variant was 4.8%. The MAF of all variants were less than 0.002 in gnomAD, 1000Genomes and EXAC databases. There are two variants that were identified in two subjects of African ancestry, the rest were present in Caucasian subjects. One variant, p.R89P, is present in 1% of the African population, the other variant, p.S246T, was present in 1000 Genomes with a frequency of 0.00019. We did not screen a control African DNA panel as it was not available to us. The rest of the variants in *SPATA13* were screened in a control cohort of 192 random European Caucasians. In this control cohort all the 13 exons of *SPATA13* were screened by Sanger sequencing where we identified one subject carrying the c.C497T, p.P166L variant. This variant also had a CADD value lower than 10 so presumably this was a rare polymorphism. This shows that the rare variants identified in this study are significantly enriched in our PAC/PACG cohort (**S4 Text**).

**Table 1:** List of variants identified in SPATA13 in a cohort of 189 PAC/PACG patients.

patient ID	Location	CDS position	Protein position	SIFT	PolyPhen	CADD score	gnomAD Allele Frequency
6:I	13:24223195–24223195	c.G266C	p.R89P	deleterious	Probably damaging	26.5	0.001335
7:I	13:24223426–24223426	c.C497T	p.P166L	tolerated	benign	9.475	0.001762
8:I	13:24223666–24223666	c.G737C	p.S246T	tolerated	benign	12.47	0.0000381
4:I	13:24223804–24223804	c.G875T	p.S292I	tolerated	possibly damaging	22.3	0.00000661
10:I	13:24224016–24224016	c.C1087T	p.R363C	deleterious	Possibly damaging	25.5	0.0002
9:I	13:24224083–24224083	c.C1154T	p.A385V	tolerated	benign	15.17	0.00115
2:II:I	13:24224347–24224347	c.G1418A	p.S473N	deleterious	Possibly damaging	22.4	0.000824
3:I	13:24290695–24290695	c.C2891T	p.P964L	tolerated	Probably damaging	24.9	0.0000244

The degree of conservation of all the variants in SPATA13 identified in this study is shown for all nine species (**Fig 3B**). The predicted secondary structure of SPATA13 shows that the first 800 residues at the N-terminus, where most of the variants are located, has higher percentage of random coil compared with the C-terminus (**Fig 3C**). The 9 base pair deletion makes the N terminus of SPATA13 more compact when compared with the wildtype protein (**compare Fig 3D with 3E**).

### Expression of SPATA13 transcripts in human cell lines and eye tissues

**Fig 4:**
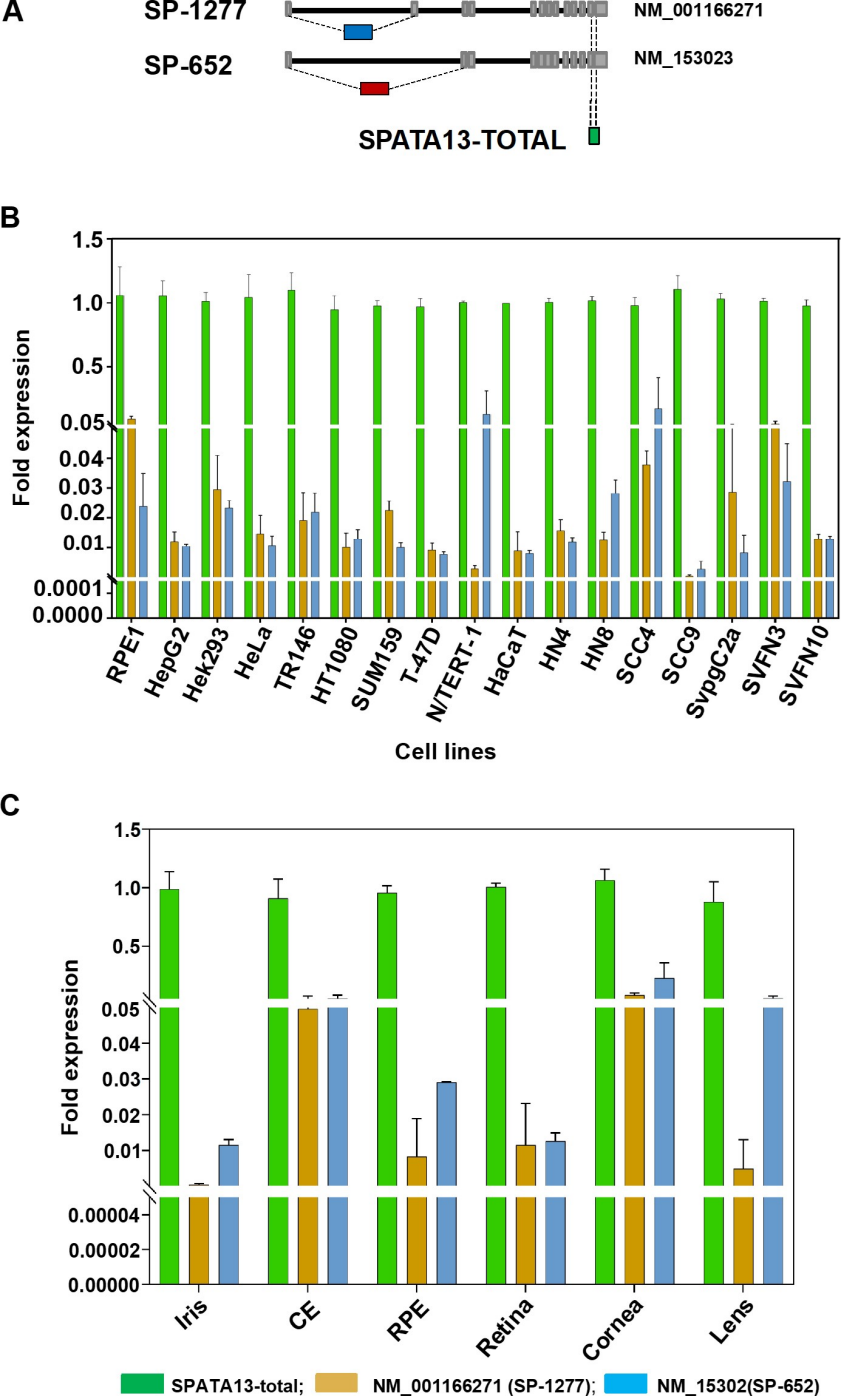
Expression of different *SPATA13* transcripts in human cell lines and eye tissues. (A) Schematic representation of the transcripts of different isoforms of SPATA13. The two isoform SP-1277 and SP-652 isoforms are generated by alternative splicing of exons toward the N-terminal. The position of qPCR primers used to differentiate the 2 transcripts are also shown. (B) Expression of SPATA13 transcripts by qPCR in 17 different cell lines derived from human eye, breast, liver, skin, head and neck, cervix and kidney. (C) Expression of SP-1277 and SP-652 transcripts in human iris, ciliary epithelium, retinal pigment epithelium, retina, cornea and lens. The green bars represent SPATA13-Total whereas the blue bars represent SP-1277 and the red bars represent SP-652.

Multiple *SPATA13* transcripts differing in their 5’ region have been reported and designated ASEF2a-d [18]. One of the short transcripts, ASEF2b (NM_153023), encoding a 652 amino acid protein (NP_694568), has been studied in detail [19–21]. This transcript also contains one of the nine mutations reported above. For simplicity, we will refer NP_001159743 as SP-1277 and NP_694568 as SP-652 based on the amino acid number they encode. We investigated the expression levels of these two SPATA13 transcripts by qPCR. Transcript specific primers were designed to detect NM_153023 (encoding SP-652) and NM_001166271 (encoding SP-1277) as well as total SPATA13 as shown in **Fig 4A**. Seventeen different human cell lines derived from the eye (retinal pigment epithelial, RPE-1), liver (HepG2), kidney (HEK293), cervix (HeLa), oesophagus (TR146), fibrosarcoma (HT1080), breast (SUM159, T47D), skin (N/Tert-1, HaCaT), head and neck cancers (HN4, HN8, SCC4, SCC9), oral dysplasia (SVpgC2a), and those produced from SVpgC2a by nicotine treatment (SVFN3 and SVFN10) [22] were analysed. As shown in **Fig 4B**, these transcripts were detected in all 17 cell lines. The transcripts for SP-1277 showed highest expression in RPE-1, SVFN3 and low in SCC9 whereas the transcript for SP-652 was highest in N/Tert-1, SCC4 and lowest in SCC9 (**Fig 4B**). These results suggest that the two transcripts are ubiquitously expressed at varying levels in multiple cell lines derived from different human tissues.

We also used qPCR to investigate the mRNA expression of SP-1277 and SP-652 in human iris, ciliary epithelium, retinal pigmented epithelium (RPE), retina, cornea and the lens (**Fig 4C**). The transcripts for SP-652 showed highest expression in cornea and lens, whereas the transcripts for SP-1277 was highest in cornea and ciliary epithelium, suggesting that SP-1277 was the predominant transcript in tissues most affected in PAC/PACG.

### SPATA13 antibodies characterisation and expression in the eye

**Fig 5:**
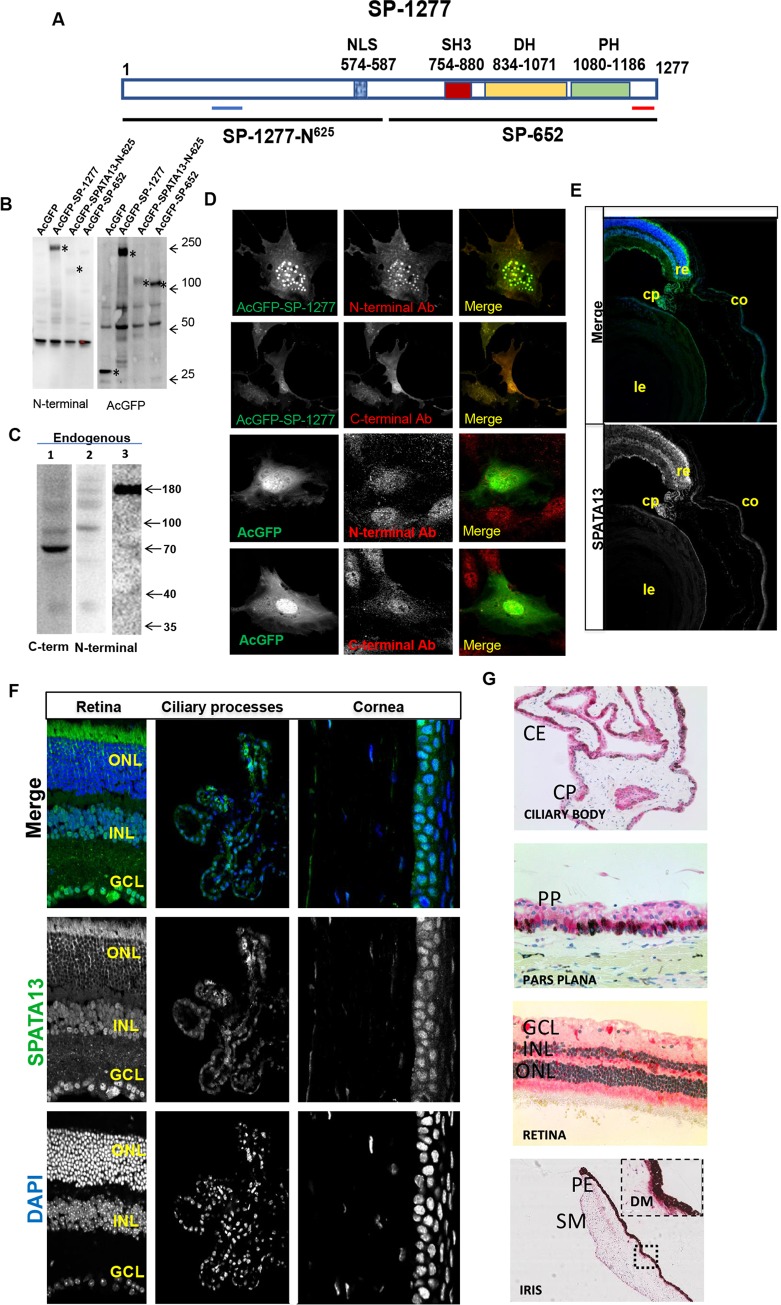
Characterisation of SP-1277 isoform. (A) Schematic representation of the domain structure of SP-1277 isoform. Light blue bar shows the epitope for the N-terminal antibodies and the red bar shows epitope for the C-terminal antibodies. The nuclear localisation signal in the terminal region SP-1277N^625^is shown by a hashed blue bar. The boundary of SP-1277-N^625^ and SP-652 are shown two discontinuous lines. The SH3, DBL homology (DH) and PH domains are shown in SP-652. (B) Western blot analysis of the SPATA13 and its fragments analysed by N-terminus (ab122627) and anti-AcGFP antibodies. AcGFP fusion protein of SP-1277, SP-1277-N^625^ and the SP-652 were expressed in HT1080 cells, total cell lysate was separated on 4–15% SDS polyacrylamide gel, transferred on nitrocellulose membrane and probed with different antibodies. Specific reactivity with protein bands is shown by asterisks. (C) Western blot of total RPE-1 lysate probed with the C-terminus (lane 1) and N-terminal (lane 2, ThermoFisher; lane 3, Abcam) antibodies. (D) Reactivity of N- and C-terminal antibodies with RPE-1 cells overexpressing SP-1277 compared with AcGFP control. Immuohistochemical reactivity of murine eye tissues (E, F) and human eye tissues (G) with the N-terminus antibody from Abcam (ab122627). CE = ciliary epithelium, CP = ciliary process, PP = pars plana, GCL = ganglion cell layer, INL = inner nuclear layer, ONL = outer nuclear layer, SM = sphincter muscle, DM = dilator muscle, PE = posterior muscle. Re = retina, Co = cornea, Le = lens.

There are several commercially available antibodies which recognise SP-1277 and SP-652. To confirm their specificities, we cloned SP-1277, SP-652 isoforms and the first N-terminal 625 residues of SP-1277 (SP-1277-N^625^) (**Fig 5A**) as AcGFP fusion proteins and expressed them in HT1080 cells. Protein bands of around 166kDa (SP-1277), 100kDa (SP-652 and SP-1277-N^625^) were detected with anti-AcGFP antibody (**Fig 5B**). In this study we have used four different antibodies, two against the N- and two against the C-terminal of SP-1277 (available from Abcam and ThermoFisher Scientific). The N-terminal antibodies recognised SP-1277 but showed no reactivity with SP-652 (representative blots of Abcam antibodies are shown in **Fig 5B**). As shown in **Fig 5C**, the Abcam C-terminal antibody recognised a band of 70kDa and several high molecular weight bands in the range of 80-180kDa (**Fig 5C, lane 1**). The N-terminal antibody from ThermoFisher reacted with several high molecular weight bands in the range of 80-180kDa (**Fig 5C, lane 2**) while the Abcam antibody recognised a single band of 180kDa (**Fig 5C, lane 3**) but did not bind to the 70kDa band showing the specificity of these reagents. To further test the specificity of the antibody on tissue culture cells, RPE-1 cells were transfected with AcGFP tagged SP-1277 (AcGFP-SP-1277) and immunostained with the N-terminal SPATA13 antibody. As shown in **Fig 5D** there was complete co-localisation of the AcGFP-SP-1277 with the N-terminal antibody. Similar results were obtained when the C-terminal SPATA13 antibody was used to identify the AcGFP-SP-1277. These results show that the N and C-terminal SPATA13 antibodies were able to recognise SP-1277 in western blots and immunostaining. In this study, the two N-terminal SPATA13 antibodies were used interchangeably, however, the data presented here are mostly with the Abcam antibody.

To investigate whether SP-1277 protein is expressed in the eye we used the N-terminus antibody on murine (**Fig 5E & 5F**) and human (**Fig 5G**) eye sections using immunohistochemistry. As shown in **Fig 5E–5G**, we observed specific reactivity with both pigmented and non-pigmented ciliary epithelia, iris sphincter and dilator muscles, corneal epithelium and retinal outer nuclear, inner nuclear and ganglion cell layers.

### SPATA13 shows nuclear and cytoplasmic localisation in RPE-1 cells

**Fig 6:**
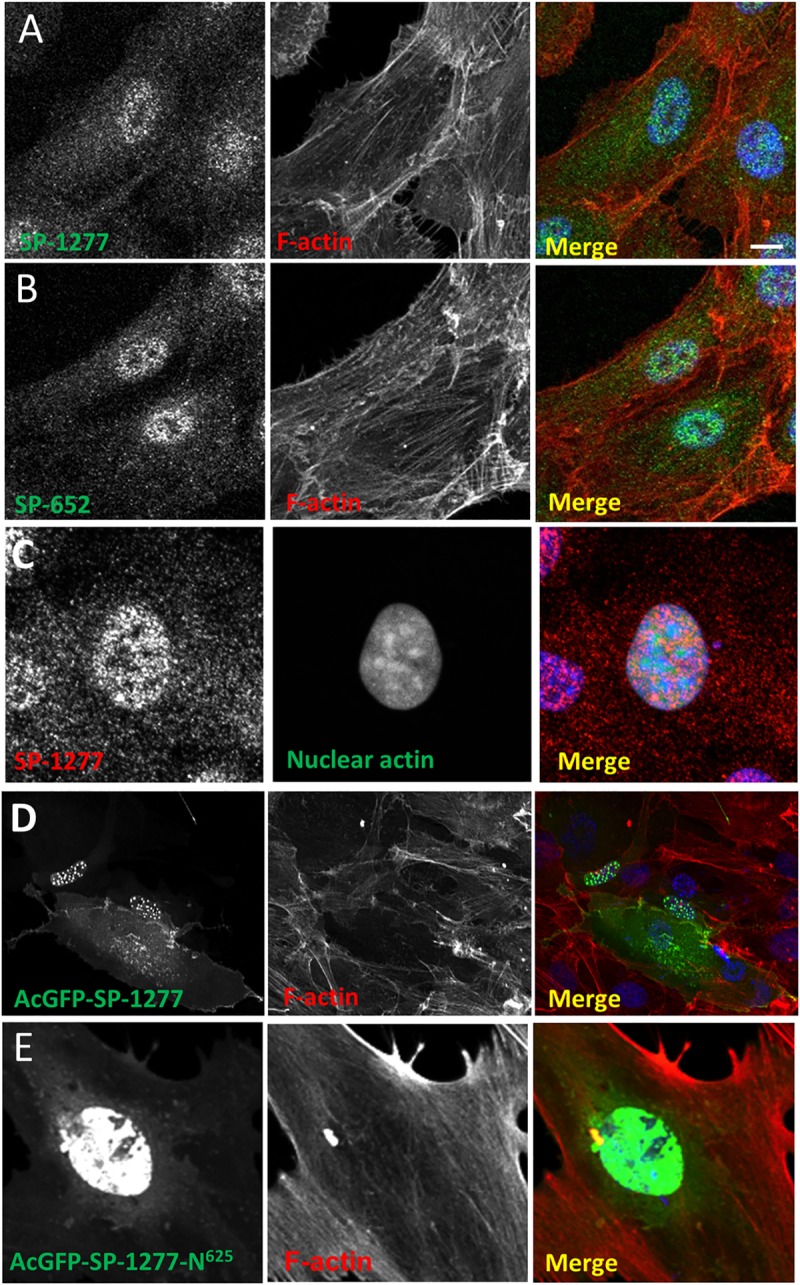
Endogenous and ectopic expression of SPATA13 isoforms. Human RPE-1 cells were immunostained with SP-1277 (N-terminus antibody, A) and SP-652 (C-terminus antibody, B) and co-stained for endogenous F-actin (Alexa Fluor 594 conjugated phalloidin). (C) To detect nuclear actin, RPE-1 cells were transfected with nuclear actin chromobody (nAC) probe construct [55] and were counterstained with N-terminal specific SP-1277 antibody. RPE-1 cells were transiently transfected with AcGFP-tagged SP-1277 (D), and SP-1277-N^625^(E) respectively, both counterstained for F-actin. AcGFP-SP-1277 localised to nuclear speckles with a diffuse but variable cytoplasmic signal (S), AcGFP-SPATA13-N^625^ (E) only showed nuclear staining. Nuclei were stained with DAPI (blue). Scale bar = 10 μM.

Having established expression of SPATA13 in the eye, we investigated the cellular localisation of the endogenous SP-1277 isoform using the antibodies characterised in **Fig 5**. With the N-terminus antibody, a predominantly grainy nuclear staining was observed in RPE-1 cells (**Fig 6A**). The C-terminus antibody, which should recognise all the SPATA13 isoforms, also gave almost identical staining (**Fig 6B**). Similar observations have been reported on HeLa cells using SPATA13 antibodies [23]. As SP-652 has been implicated in actin function [23], we investigated the co-localisation of SPATA13 with F-actin in RPE-1 cells. **Fig 6A and 6B** show that both the N- and C-terminal antibodies gave reactivity in the nucleus as well as in the cytoplasm with little co-localisation with F-actin. We also observed no co-localisation of SPATA13 staining with nuclear actin (**Fig 6C**).

To investigate the functions of each isoform individually, we transfected AcGFP-SP-1277 and its N-terminus 625 residues (AcGFP-SP-1277-N^625^) in RPE-1 cells. AcGFP-SP-1277 was primarily localised as globules in the nucleus with some cytoplasmic staining (**Fig 6D**), which could be due to a single 14 residues bipartite nuclear localisation signal (NLS) 574-RTPKRRWGSGRRPR-587 identified by NLStradamus [24] (**Fig 5A**). When AcGFP-SP-1277-N^625^ was ectopically expressed in RPE-1 cells, it gave homogenous nuclear AcGFP expression (**Fig 6E**). Four of the nine variants (S292I, S473N, 478-480d and P964L) reported here were introduced by site-directed mutagenesis and expressed in RPE-1 cells. The nuclear localisation of these variants did not change when compared with the wildtype suggesting the mutations do not affect localisation. (**S1 Fig**).

### Role of SP-1277 in mitosis

**Fig 7:**
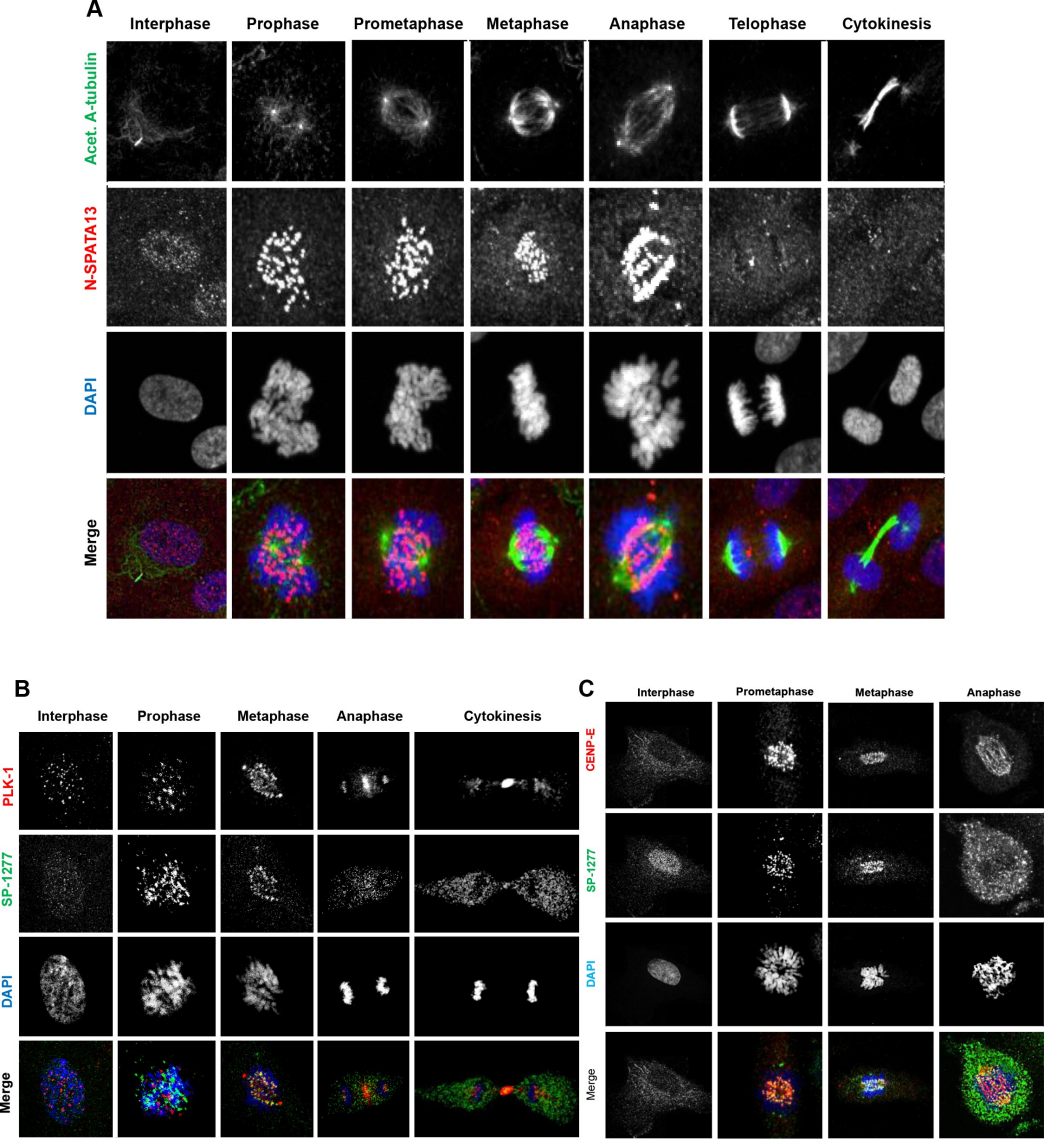
Endogenous SP-1277 isoform during mitosis. Endogenous SP-1277 protein was localised in dividing RPE-1 cells, during mitosis using the N-terminus antibody and counterstained using anti-acetylated ⍺-tubulin antibody (A), or counter stained with two mitotic markers PLK-1 (B) and CENP-E (C). (A) SP-1277 was detected in the nucleus as speckles at G0, which intensified at the onset of prophase, aligning along the kinetochore at metaphase, and anaphase, before returning to its G0 localisation pattern. (B) SP-1277 globules show co-localisation with the kinetochore marker PLK-1 at prophase, metaphase and anaphase. At cytokinesis the PLK-1 staining is primarily localized at midbody between the dividing cell which also co-stains for SP-1277. (C) SP-1277 show strong co-localisation with another kinetochore marker CENP-E at prophase, prometaphase and anaphase. At anaphase the colocalization primarily is around the separated chromosomes. Nuclei were stained using DAPI (blue).

While investigating the cellular expression of SPATA13 isoforms, we observed that some cells undergoing cell division showed very speckled localisation of SP-1277 in the equatorial region of the dividing cells. To investigate this further, we examined the localisation of SP-1277 in RPE-1 cells at different stages of cell division (**Fig 7**). SP-1277 showed granular nuclear signal at interphase with a faint background cytoplasmic signal which remained visible throughout mitosis (**Fig 7A**). Cells were co-stained with acetylated ⍺-tubulin, to mark the microtubules, centrosomes and spindles. At the beginning of prophase, SP-1277 coalesced into intense speckles within the nucleus, which condensed further and moved towards the equatorial region during metaphase. At anaphase, SP-1277 speckles localised specifically along the kinetochore. The SP-1277 signal dispersed as the daughter cells separated, with only a faint background signal remaining.

**Fig 8:**
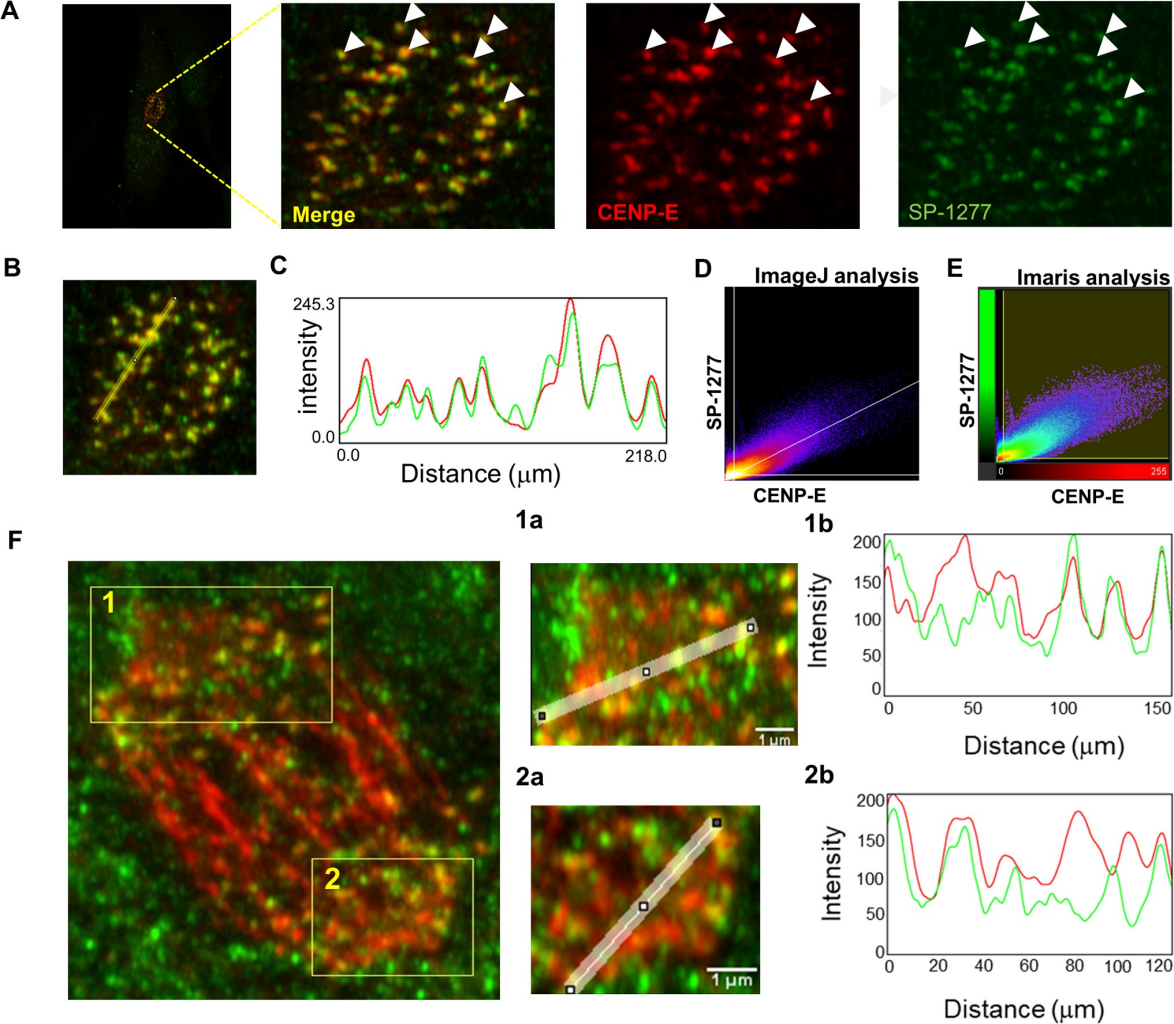
Colocalization analysis of SP-1277 with CENP-E at prometaphase and anaphase. (A) At prometaphase SP-1277 (green) co-localises with CENPE-E (red) globules. To evaluate whether colocalization occurs, a line was drawn connecting different globules (B). The green and red intensity was measured using ImageJ in globules along the line and plotted (C). The data was analysed using Image J and Imaris softwares and showed colocalization of 84% (tM0.84) of CENP-E with SP-1277 and 75% (tM = 0.75) of SP-1277 with CENP-E. Fluorescence intensity data of two images were distributed linearly as shown in the scatterplots (Fig 8D & 8E). The colocalization of SP-1277 with CENP-E in anaphase is shown in (F). The regions of interest showing highest colocalization are labelled as 1 and 2. The degree of colocalization of the two molecules was analysed using ImageJ (1a, and 1b for region 1) and (2a and 2b for region 2) and gave a colocalization of more than 80%.

To investigate this novel observation, we examined whether SP-1277 was colocalising with the kinetochore. We co-stained RPE-1 cells for SP-1277 and two kinetochore markers, polo-like kinase 1 (PLK-1) and centrosome associated protein E (CENP-E). As shown in **Fig 7B**, SP-1277 starts to co-localise with PLK-1 at the beginning of prophase and remains co-localised along the kinetochore complex in pro-metaphase and metaphase. At cytokinesis SP-1277 distributes in the cytoplasm with strong staining at the midbody. Similar colocalisation of SP-1277 was observed with CENP-E (**Fig 7C**). To quantify the degree of colocalisation we selected RPE-1 at two different stages of mitosis, prophase (**Fig 8A**) and anaphase (**Fig 8F**). Visual methods (described in **S5 Text**) indicated colocalisation of SP-1277 (green) and CENP-E (red) at prophase as shown in **Fig 8A** and in **Fig 8B** and **8C**. We also observed that the colocalisation showed strong linear distribution in the scatterplots (**Fig 8D and 8E**). Automatic thresholding algorithm of Costes [25] showed localisation of CENP-E was 84% with SP-1277 and 75% of SP1277 localised with CENP-E. Similar results were obtained with ImageJ colocalisation plugins and the image analysis software package Imaris (**Fig 8D & 8E**). At anaphase SP-1277 and CENP-E showed 84% colocalisation (**Fig 8F**), with more colocalisation at the kinetochores of the separating chromosome and very little at the metaphase plate.

### Influence of mutations on the GEF activity of SP-1277

**Fig 9:**
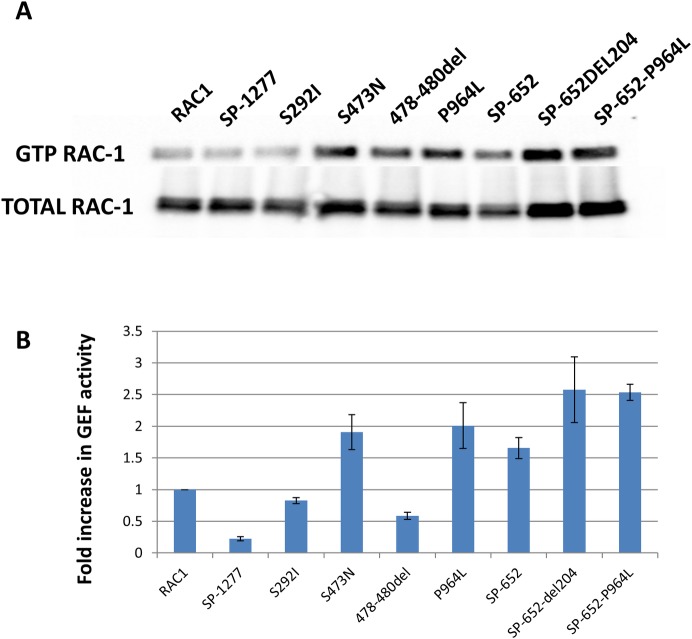
SPATA13 mutations induced GEF activity in SP-1277. (A) RPE-1 cells were co-transfected with Flag-RAC-1 along with wildtype SP-1277, wildtype SP-652 or mutants (S292I, S473N, 478-480d, P964L) cloned in pLPCpuro_NAcGFP and after lysis the GTP-RAC-1 was immunoprecipitated with PAK-PBD beads and analysed on western blot using anti-Flag antibody. (B) Quantification of GEF activity of SP-1277 (wildtype and mutants). One way ANOVA was used to test the null hypothesis of no difference between GEF activity in mutants and wildtype, with there being a significant difference (p = 0.002).

As SP-652 has been shown to have GEF activity, we investigated if additional 625 residues at the N-terminus of SP-652 would affect this activity. GEF activity was measured by co-transfecting AcGFP-SP-1277 (wildtype or mutants) and RAC-1 in RPE-1 cells (**Fig 9**). In these experiments, SP-652 lacking the first 204 residues (SP-652del204) was used as positive control as it has been shown that removing 204 residues from the N-terminus increases its GEF activity [26]. There was a 1.5 fold increase in GEF activity of SP-652 compared with RAC-1 only, which increased to 2.5 fold when the first 204 residues were deleted.

SP-1277 had lower GEF activity than SP-652 (**Fig 9**) suggesting the random coil region in SP-1277 had a negative impact on its GEF activity. We also investigated whether the 9 bp deletion affected the GEF activity of SP-1277. There was more than 2 fold increase in GEF activity due to the 9 bp deletion. We further investigated the GEF activity in another three of the nine variants (S292I, S473N, P964L). The highest change was observed in S473N and P964L. The activity of SP-652 containing P964L mutation was also increased by about 2.5 fold suggesting that this mutation clearly affected the GEF activity.

### Discussion

Pathogenesis of angle closure glaucoma is complex with multiple heritable factors affecting the disease progression and severity. Singling out individual factors contributing to the disease is crucial for unravelling the pathway leading to the development of PACG. The lack of suitably large families with multiple affected individuals, together with disease heterogeneity, has hampered classical genetic linkage analyses and positional mapping studies of PACG. However, recent advances in medical imaging techniques, such as anterior segment optical coherence tomography (AS-OCT) have improved phenotypic classification of the angle-closure disease. We used this technique to supplement clinical examination of the relationship between the angle and iris and lens.

In this study, we have used detailed phenotyping in combination with linkage analysis of the largest reported PACG family and next generation sequencing to identify *SPATA13/ASEF2* as a gene that harbors a disease-causing mutation leading to PACG. In this family PACG was associated with a 9 bp deletion in *SPATA13* showing variable expression and reduced penetrance (**S6 Text**). There were 39 blood related subjects that were clinically examined and genotyped, of these 5 were unaffected, giving us a reduced penetrance of 87.2%. However, of the 5 unaffected, 2 males, VI:25 (42y) and VI:26 (48y), may develop PAC/PACG later, so excluding them from the analysis would increase the penetrance to 92.3%. There is a possibility, although very small, that there might be another variant deep in the intron or in another gene in linkage disequilibrium with *SPATA13* as the filtration of variants were prioritised for coding regions. However, we identified additional 8 individuals with rare variants in *SPATA13*, one of whom carried the same 9 bp deletion, strengthening the genetic evidence (**Table 1**). Analysis of SNPs within *SPATA1*3 showed that the second proband with the 9 bp deletion is not related to Family 1. In this family the penetrance appears to be low, but we believe that the phenotype of the daughter is inherited from her mother, giving a penetrance of 100%. This is explained in detail in **S3 Text**.

Further evidence for the involvement of *SPATA13* in PACG comes from the protein expression data shown in **Fig 5**. This is the first study where SP-1277 expression is shown in iris, cornea, ciliary epithelium and retina of the human and murine eyes, the tissues most affected in PACG.

A number of GWAS studies have implicated SP-1277 in several neurological disorders including intellectual disability [27], comorbid depression syndrome and alcohol dependence [28] and anorexia nervosa in human [29] and social hierarchy and nocturnal activity in mice [30]. It has also been implicated in differentiated thyroid cancer risk [31] and BRCA1-like early onset breast cancer [32]. The SPATA13 gene product has never been implicated in any eye disease, this is the first study implicating this protein in PACG. The gene produces several transcripts, here we have shown that two *SPATA13* transcripts are ubiquitously present in cell lines derived from different human tissues as well as in the eye tissues including cornea, iris, retina, RPE and the lens. This raises an interesting question; if SPATA13 is a ubiquitously expressed protein, why do mutations in this gene only affect the eye? The obvious possibility is that the mutations do cause pathogenesis in other tissues, but they have not been diagnosed or investigated. It is also conceivable that in other organs the effect of mutations is overcome by tissue-specific expression of other proteins. However, there are reports where mutations in a ubiquitously expressed protein only affects the eye, e.g., mutations in *PRPF31* only cause retinitis pigmentosa and does not affect any other part of the body [33].

The SP-652 isoform has been reported to regulate cellular events such as reorganisation of cytoskeletal structures, cell migration [23] and angiogenesis [34, 35]. The other isoform SP-1277 which has an additional 625 residues at the N-terminus due to alternative splicing of SP-652, has never been investigated and its function is not known. SP-652 contains an adenomatous polyposis coli (APC)-binding region (ABR) followed by a Src homology 3 (SH3) domain, a Dbl homology (DH) domain and a Pleckstrin (PH) domain (Fig 5A), which is also present in SP-1277. The ABR-SH3 domain binds to APC, which activates its GEF activity to mediate downstream cellular events [18]. At the protein level we show that endogenous SP-1277 localises to both nucleus and cytoplasm in interphase, which is consistent with previous reports for endogenous SP-652 expression [36]. In silico 3-D structural prediction analysis showed that SP-1277 contains 16 intrinsically disordered regions (IDR) (**Fig 3C**), the longest of which is 157 residues. It has been reported that SP-652 is normally in an inactive conformation until the Armadillo (ARM) domain of APC binds to the ABR and SH3 domain and facilitate the Cdc42 exchange [26]. It is likely that post translational modification or binding of APC to the ABR-SH3 domain of SP-1277 releases the DH domain activating its GEF activity and that the IDR acquires a tertiary structure creating binding sites for other proteins and/or DNA/RNA for its additional downstream functions. SP-652 lacks this IDR and therefore binding of APC will result in downstream functions different to SP-1277. The 9 bp deletion (p.478_480del) is predicted to make the IDRs more compact (**compare Fig 3D with 3E**), which would likely affect their interactions with binding proteins including APC.

In this study we have shown dramatic redistribution of SP-1277 during different stages of cell division suggesting a role in mitosis. Although there are fundamental anatomical and structural differences between the mouse and human eyes, such as lack of a lamina cribrosa or a macula, which may lead to different neurodegenerative pathways, two independent studies have shown that complete knockout of all SPATA13 isoforms in mouse was not embryonic lethal and had no ocular phenotype [30, 34]. This suggests that SPATA13 was not essential for mitosis, but instead may have a regulatory role. Similar results have been reported with myocilin (*MYOC*), a gene associated with POAG pathogenesis [37]. Heterozygous and homozygous mice lacking myocilin show no ocular phenotype, however, missense mutations in this protein are not tolerated and lead to POAG [38].

Rho GTPases are involved in major cellular functions including cell growth, adhesion, motility, polarity and differentiation by controlling different cellular processes such as cytoskeletal remodeling, microtubule dynamics, gene transcription and phospholipid metabolism. Rho signaling responses in cells are highly regulated spatiotemporally to ensure homeostasis within the cell. They switch between active GTP bound and inactive GDP bound states with the help of RHO-GEFs, RHO-GAPs and RHO-guanine nucleotide dissociation inhibitors (RHO-GDIs). The localised modulation of Rho GTPases by GEFs, GAPs and GDIs depends on cell type and presents a complex network of highly integrated regulatory mechanisms. Rho GTPases are also key components of neuronal cell degeneration pathways [39]. The Rho GTPase signaling pathway has been shown to modulate aqueous humour outflow by regulating the contractile properties of trabecular meshwork and Schlemm’s canal cells in humans [40, 41]. Inactivation of Rho GTPases significantly stimulated/increased adult ciliary epithelial cell proliferation *in situ* [42]. One of the key features of RHO-GEFs and RHO-GAPs is that their activities are autoinhibited and are sensitive to local activation. SP-652 has been shown to be inactive for RHO-GEF and is activated by binding to APC. In this report we only investigated 4 of the 8 SPATA13 variants for their effect on GEF activity. We have shown that SP-1277 exhibits less GEF activity than SP-652 and the 9bp deletion increases the RAC1-dependent GEF activity. This has also been observed for three additional variants identified in *SPATA13* (**Fig 9**), which suggested that conformational changes introduced by mutations in these variants affect the function through altering the GEF activity. It is likely that the rest of the variants that have not been tested might behave differently and may not show any difference in their GEF activity. It should be noted that PLEKHA7, another protein implicated in PACG (19), has GAP activity and therefore could interact with RAC1 similar to SP-1277. It is, therefore, also likely that both SP-1277 and PLEKHA7 are involved in Rho-GTPase pathway, indicating this pathway could play an important role in PACG disease pathogenesis.

It is important to note that a proportion of SP-1277 is always in the cytoplasm even in dividing cells suggesting that it can also play a role in cell adhesion and migration. The mutations reported here enhance GEF activity of SP-1277, which could dysregulate mitosis with impact on symmetric/asymmetric cell division, cell adhesion and migration as has been reported for SP-652 [19, 21, 23, 43, 44]. Dysfunction in cell adhesion has been identified as a genetically modulated risk factor in PACG (14–19) and is likely to cause its effect through degradation of the blood aqueous barrier. PLEKHA7 encodes an apical junctional protein that is expressed in the non-pigmented ciliary epithelium, a key component of the blood aqueous barrier (BAB). Lee *et al* found that PLEKHA7 is down regulated in the iris of PACG patients that carry the C risk allele at SNP 11024102[13]. Silencing of PLEKHA7 in non-pigmented ciliary epithelium (NPCE) affected actin cytoskeleton organization *in vitro*. Consistent with the regulatory role of RAC-1 and Cdc42 in maintaining tight junction permeability, silencing of PLEKHA7 compromises the paracellular barrier between NPCE cells *in vitro*. The authors concluded that downregulation of PLEKHA7 in PACG may affect BAB integrity [13].

Other evidence supporting the involvement of SPATA13 in PACG comes from our expression studies where it is highly expressed in iris, cornea, ciliary body and retina (**Fig 5C–5F**), the tissue most affected by the disease. The malformation of the ciliary epithelium would also impact aqueous humour secretion as well as attachment of lens zonules which would affect the positioning of the lens as observed in PACG [45]. Disturbances in tissue homeostasis of the uvea may increase the propensity for shedding of pigment from the pigmented ciliary or iris epithelia. We observed increased trabecular meshwork pigmentation in all patients carrying the 9 bp deletion, except the youngest two males aged 24y and 26y, in whom disease signs might develop later.

APC and the kinetochore associated proteins have been investigated as targets for developing therapeutics for cancer treatment [46]. APC, which interacts with the two close homologues, ASEF1 and ASEF2 (41), is a peptidomimetic target for colon cancer treatment [47]. Cell cycle inhibitors are being investigated in targeting preferentially aneuploid cancer cells while sparing normal diploid cells. Similar strategies may offer the prospect of treatment for PACG involving APC-SPATA13/ASEF2 interactions.

In conclusion, we have identified *SPATA13* as a gene in which mutations cause primary angle-closure disease, which in turn puts those affected at risk of developing glaucomatous loss of vision. There is variable expression and incomplete penetrance of the phenotype associated with this gene. Detailed functional characterisation of SP-1277 and mutations identified in this study will provide insights into the mechanism of disease pathogenesis, strengthening the hypothesis that abnormalities in cell division and/or cell adhesion in the anterior segment of the eye might be a common mechanism in the development of PACG. Identification of *SPATA13* as one of the disease-causing genes for PACG opens new avenues for identifying additional genes in this pathway causing PACG.

### Methods and materials

Informed, written consent was obtained from all participants. This study followed the principles of the Declaration of Helsinki. The study was approved by the Moorfields NHS Research Ethics Committee (06/Q0504/8).

#### Family history and pedigree recruitment

The index patient for each family (proband), underwent pedigree charting, enquiring about a family history of glaucoma and/or angle-closure. The accuracy of family history information was confirmed by corroboration of relatives attending the clinics. All family members of the proband, aged 20 years and older were invited to attend ophthalmic examinations. The Society of Genealogists (http://www.sog.org.uk/) helped identify distant relatives using publicly available genealogy and census data. A family member from of the five largest families sent letters of invitation explaining the research to their distant relatives, inviting them to participate.

#### Clinical examination

Detail clinical examination procedure and diagnostic classification is given in **S2 Text.** Each participant examined in research clinics underwent comprehensive ophthalmic examination including dark room gonioscopy, visual field testing as well as a clinical examination of the optic disc, and supplementary imaging of the optic disc and peripapillary retina. The primary defining feature of cases was contact between the iris and trabecular meshwork identified on dark room gonioscopy and/or anterior segment imaging. Primary angle-closure glaucoma was diagnosed when there was evidence of iridotrabecular contact and glaucomatous damage to the optic nerve, as detailed under the ISGEO diagnostic standards [2].

#### DNA isolation and Sanger sequencing

DNA was extracted from peripheral blood using Gentra PureGene kit (Qiagen, UK). Sanger sequencing was performed with BigDyev3.1 (Life Technologies) on ABI3730 according to manufacturer’s protocol. 192 human random control DNAs were purchased from Merk.

#### SNP chip array and linkage analysis

Single nucleotide polymorphism (SNP) genotyping was undertaken on 11 individuals from Family I. Processing of SNP microarrays (Illumina, Human CytoSNP-12, 298,199 markers) was performed according to the manufacturer's protocol. The relationship status of participants was checked against their genotype using the GRR software (http://csg.sph.umich.edu/abecasis/grr/) to identify errors in pedigree charting and possible non-paternity. Overall call rates were >98% for all samples and no gender mismatch was found. The marker sets were reduced to the 3,547 most informative markers distributed uniformly throughout the chromosomes to avoid artificially inflating LOD scores due to linkage disequilibrium between closely spaced SNPs. All genotype calls were checked for Mendelian inconsistencies and two-point and multipoint linkage analyses of genome wide SNP data were performed using Superlink-Online SNP-1.1 [48] using an autosomal dominant model, 100% penetrance in the affected-only analysis, 90% penetrance in other analyses.

#### Exome and whole genome sequencing

Genomic DNA from the proband of family I was fragmented enriched for exomic sequences using the Agilent SureSelect Whole Exome hybrid capture (Agilent, UK). The resulting enriched sequence library was sequenced with 76bp paired end reads across two lanes of Illumina GAIIx flowcell (v2 chemistry). The sequence reads were aligned to the reference genome (hg18) with the Novoalign aligner. Duplicate reads, resulting from PCR clonality or optical duplicates, and reads mapping to multiple locations were excluded from downstream analysis. Single nucleotide substitutions and small insertion/deletions were identified, and quality filtered within the SamTools software package.

Whole genome sequencing was performed on HiSeqX with 30x coverage at Edinburgh Genomic Centre, UK. The sequence reads were aligned to hg19 and the data was obtained as BAM and VCF files. VCFTools were used to filter the variants within the genetically linked region. Pathogenicity of the variants were evaluated by VEF (Ensemble) and CADD.

#### Antibodies

Two SPATA13 antibodies, SP-1277 (ab122627) and SP-652 (ab122701) were purchased from Abcam (UK), PA5-59479, PA5-59096 from Thermofisher antibodies, PLK-1 (ab17057) and CENP-E (ab5093) from Abcam. Mouse monoclonal alpha-tubulin antibody (ab7291), were purchased from Abcam (UK). Alexa Fluor 488-conjugated goat anti-mouse (A11001) and anti-rabbit (A11008) antibodies, Alexa Fluor 594-conjugated goat anti-mouse (A11005) and anti-rabbit (A11034) antibodies, and donkey anti-goat antibodies conjugated with Alexa Fluor 488 (A11055) and with Alexa Fluor 594 (A11058), were purchased from Life Technologies (CA, USA). Alexa Fluor 594 labelled Phalloidin was purchased from Life Technologies (CA, USA). Horseradish peroxidase-conjugated goat anti-rabbit and anti-mouse IgG were purchased from Jackson Immuno Research Laboratories, Inc. (PA, USA).

#### Molecular cloning and mutagenesis

Total mRNA was isolated from cultured cells using the Dynabeads mRNA DIRECT Purification Kit (Ambion, Paisley, UK) using manufacturer’s instructions and transcribed into cDNA using the Transcriptor Reverse Transcriptase System (Roche, Burgess Hill, UK). The coding regions of SP-652 and SP-1277 were amplified using high fidelity Q5 DNA polymerase (New England Biolabs, UK) and ligated into pLPC_NAcGFP using standard cloning techniques [49]. The retroviral expression vector pLPC_NMyc (gift from Professor Titia de Lange (Addgene plasmid #12540) was re-engineered to replace N-Myc tag with AcGFP. For site-directed mutagenesis phosphorylated primers harbouring the desired mutations were used to amplify the SPATA13 constructs with Q5 DNA polymerase and ligated with Taq DNA ligase. Sanger sequencing was used to verify all cloned inserts and mutations.

#### SDS electrophoresis and western blotting

SDS electrophoresis and western blotting were performed according to protocol described previously [50].

#### mRNA extraction, cDNA synthesis and quantitative PCR

Total RNAs from pooled human eye tissues, ciliary body, iris, retinal epithelium and retina were commercially obtained from 3H Biomedical AB (Sweden) and isolated from cornea and lens tissues using RNeasy Mini Kit (Qiagen) as per manufacturer’s instructions. The RNA samples were reverse transcribed into cDNAs as described above. Specific primers for three different forms of *SPATA13* (NM_001286792; NM_001166271 and NM_153023; were designed spanning exon-exon junctions with Tm of forward and reverse primers within ±1°C. NM_001286792; F: AAGGACCCTCTGAGGACGTAG; R: ACAGGTCACCCCTAGCTGG, NM_001166271; F: ACGCTGACTTTGTAGGCTCC; R: TCATCTCCAGGAATGCCGTC, NM_153023; F: GACACGCTGACTTTGTAGGC; R: ATCCTGTGATGAGCTTCGCC, SPATA13_Total; F: GCTCAAAAGGCAGGACATGG; R: CATAGTGATGTGGCGCTGGT. Quantitative PCR (qPCR) was performed on the LightCycler 480 qPCR System (Roche, UK) according to protocol described by Bose et al. [51]. The qPCR analyses for mRNA expression in cell lines were performed in three independent replicates, with three technical repeats for every sample. However, for human eye tissues the expression was performed only in three technical replicates.

#### Mammalian Cell Culture and Immunocytochemistry

Human (h)TERT-RPE-1 retinal pigment epithelial cell line was maintained in Dulbecco's modified Eagle's medium (DMEM)/F-12+GlutaMAX (Life Technologies, CA, USA) supplemented with 10% FCS and penicillin–streptomycin (100 μg/ml). All cells were grown in 6-well plates at 1.2 x10^6^ cells per well at 37°C in an atmosphere of 5% CO_2_.

Prior to immunofluorescence (IF) studies, cells were seeded in 24-well plates on glass coverslips at 5.0 x 10^4^ cells per well and incubated for 24 h. The cells were processed for immunocytochemistry as described previously [50].

Transfections were performed in semi-confluent hTERT-RPE1 or HT1080 cells using the TransIT-LT1 Transfection Reagent (Mirus Bio LLC, USA), according to the manufacturer’s instructions. The cells were fixed 24 h post transfection prior to IF staining as described above. Retroviral packaging and transduction was carried as described previously [50]

#### Imaging and image processing

Confocal images were obtained on Zeiss LSM880 with a 63x/1.4NA oil objective. Quantitation of colocolisation is described in **S5 Text.**

#### GEF activity measurement

RPE-1 cells were co-transfected with wildtype SP-1277 or mutants (S292I, S473N, 478-480del, P964L) or wildtype SP-652 or SP-652(P964L) or SP-652del204 cloned in pLPCpuro_NAcGFP along with Flag-RAC-1 using TransIT-LT1 Transfection Reagent (Mirus Bio LLC, USA), according to the manufacturer’s instructions. After 24 h the transfected cells were starved in 0.5% serum for 4 h. The cells were lysed, GTP-RAC-1 was pulled down using PAK-PBD beads (Cytoskeleton incorporation, USA) and analysed on 4–15% gradient gel followed by western blot using anti-Flag antibody. The blots were analysed and quantified by ChemiDoc using Image Lab software from Bio-Rad.

#### Immunohistochemistry

Formalin fixed paraffin embedded human eye tissues and murine eye were cut into 5μm sections, dewaxed using xylene and immunostained using SP-1277 N-terminus specific antibody as described previously [52, 53].

#### 3D structure prediction

I-TASSER was used for prediction of secondary and tertiary structure of SPATA13 [54].

***Statistical analysis*:** Gene expression data were exported from Roche LightCycler LC480 Software as text files for subsequent analysis. Statistical analysis for qPCR and GEF assays were carried out by the t-test on Graph Pad Prism software and Microsoft Excel.

#### URLs used in the study

http://www.ensembl.org/index.html

http://csg.sph.umich.edu/abecasis/grr/

http://cbl-hap.cs.technion.ac.il/superlink-snp/

https://zhanglab.ccmb.med.umich.edu/I-TASSER/

http://cadd.gs.washington.edu/

http://www.sog.org.uk/

## Supporting information

S1 Text Selection criteria for Family 1. (DOCX)

S2 Text Clinical examination & Diagnostic Classification. (DOCX)

S3 Text SNP analysis and clinical details of family 5:II. (DOCX)

S4 Text Pathogenicity of variants. (DOCX)

S5 Text Co-localisation analysis of SP-1277 with the kinetochore markers, PLK-1 and CENPE. (DOCX)

S6 Text Variable expression and incomplete penetrance in Family 1. (DOCX)

S1 Fig Overexpression of mutant SPATA13-FL protein tagged with AcGFP. RPE-1 cells were transiently transfected with mutant AcGFP-tagged SPATA13 constructs, and co-stained using anti-SC35 antibody, a marker for nuclear speckles. Mutations are as follows: (A) SP-1277-S292I; (B) SP-1277-S473N; (C) SP-1277-9bp del; (D) SP-1277-P964L; and (E) SP-652-P964L. Mutant proteins did not show co-localisation with SC35. Nuclei were stained using DAPI (blue).
(TIF)

S1 Table Detailed clinical information of members in Family 1. (XLSX)

S2 Table Genotypes of eight affected and two unaffected subjects of Family 1. (XLSX)

S3 Table Detailed clinical information of 8 additional PAC/PACG patients and their relatives carrying mutations in *SPATA13*. (XLSX)
