# Supplementary material for: Mutations in SPATA13/ASEF2 cause primary angle closure glaucoma
Source: PLoS Genet. 2020 Apr 27;16(4):e1008721. doi: 10.1371/journal.pgen.1008721 (PMC7233598; doi:10.1371/journal.pgen.1008721)
Supplement: S1 Text — (DOCX) [file pgen.1008721.s001.docx]

**S1 Text: Selection criteria for Family 1**

Two key clinical characteristics present in the majority of affected individuals were: (1) the presence of plateau iris configuration (PIC) on gonioscopy and anterior segment optical coherence tomography imaging (AS-OCT, **Fig. 2A**), as well as (2) axial biometry that was shorter than age and sex-specific means. The complete pedigree is shown in **figure 1** and the detailed clinical phenotype of the family members examined is given in **S1 Table**. A group of healthy controls (n=795) from the European Prospective Investigation of Cancer (EPIC)-Norfolk cohort was used to calculate age and sex specific mean values for ocular biometry.
