# Supplementary material for: Mutations in SPATA13/ASEF2 cause primary angle closure glaucoma
Source: PLoS Genet. 2020 Apr 27;16(4):e1008721. doi: 10.1371/journal.pgen.1008721 (PMC7233598; doi:10.1371/journal.pgen.1008721)
Supplement: S2 Text — (DOCX) [file pgen.1008721.s002.docx]

**S2 Text: Clinical examination & Diagnostic Classification**

Each participant examined in research clinics underwent comprehensive ophthalmic slit-lamp examinations including gonioscopy, Goldmann applanation tonometry, dilated optic disc assessment with a +90D aspheric lens. Ancillary testing comprised anterior segment optical coherence tomography (AS-OCT) imaging (Visante, Carl Zeiss Meditec AG, Jena, Germany), auto-refraction (Humphrey 597K, Zeiss Humphrey, San Leonardo, CA, USA), central corneal thickness (Altair, Optikon, Rome, Italy) and 24-2 SITA fast visual field tests (Zeiss Humphrey HFA MkII visual field analyser 750i, Carl Zeiss Meditec AG, Jena, Germany) were performed. Imaging of the optic nerve head and peripapillary retinal nerve fibre layer were carried out using Heidelberg Retina Tomograph confocal scanning laser ophthalmoscope (HRT2, Heidelberg Engineering GmbH, Heidelberg, Germany) and GDx-VCC scanning laser polarimeter (Laser Diagnostic Technologies, Inc, San Diego, CA, USA). If features suggestive of glaucomatous optic neuropathy were identified on clinical examination, visual field examination (as per ISGEO criteria[1]), or optic disc imaging, 24-2 SITA standard field tests were performed. Gonioscopy was performed in dark room conditions using both magnifying two mirror gonioscopes to determine presence or absence of ITC, and four mirror gonioscopy used to identify irreversible ITC, diagnosed as peripheral anterior synechiae (PAS). A 1 mm long very narrow beam was used. This was kept well away from the pupil to prevent inadvertent pupil constriction. Slight tilting was permitted to allow a view over the convexity of the iris, in cases of a steep, convex iris profile. Plateau iris configuration (PIC) was identified in cases in which the plane of the iris made an abrupt posterior angulation in the peripheral third, and in which dynamic gonioscopy identified a “double hump”. The “double hump sign” is seen during dynamic gonioscopy when indentation of the cornea raises anterior segment pressure causing the iris to be forced posteriorly, draping over an anteriorly positioned ciliary process and over the anterior surface of the lens, and creating a trough in the lens equatorial sulcus. We classified primary angle-closure according to the ISGEO system [1], identifying three distinct stages in the natural history corresponding to

(1) PACS: an anatomically narrow angle*, with statistically normal intraocular pressure and no peripheral anterior synechiae

(2) PAC: an anatomically narrow angle, with statistically elevated intraocular pressure (> 21mmHg) and/or peripheral anterior synechiae,

(3) PACG: an anatomically narrow angle together with structural abnormality of the optic disc and a reproducible visual field abnormality, both consistent with glaucoma, with no alternative explanation.

*A narrow angle was defined as one in which the posterior, usually pigmented, trabecular meshwork was not visible for 180 degrees of angle circumference in the primary position with verified irido-trabecular contact, seen on gonioscopy and/or on anterior segment OCT.

Acutely symptomatic presentations with elevated IOP were termed APAC or APACG, depending on whether there was glaucomatous optic neuropathy (APACG implies glaucoma damage to optic nerve was seen).

Plateau iris configuration (PIC) of the peripheral iris has been observed in both sporadic and familial PACG [2]. PIC is defined anatomically as the presence of an iris root that initially rises anteriorly then, in the peripheral third, sharply angulates towards the visual axis [3]. PIC cases often have deeper anterior chambers that may appear to be of "normal" depth on clinical examination. The classification of PACG based on anatomic levels of obstruction to aqueous flow in primary and secondary angle closure glaucoma identifies four levels of progressively posterior obstructions to aqueous flow that may result in angle-closure: (i) pupil block, (ii) PIC and angle crowding; (iii) lens intumescence and (iv) retro-lenticular forces[4]. The clinical phenotype of PACG can be variable and may not be consistent within a family [5-7]. Adverse effects of therapeutic and recreational drugs as well as environmental factors increase the risk of the manifestation of angle-closure disease [8].

In selection for linkage analysis, all the “clearly affected with PACS/PAC/PACG” were selected using the criteria outlined above. Ongoing clinical care was offered to participants with established disease, or at risk of disease (i.e. PACS), in National Health Service clinics at Moorfields Eye Hospital, London.

**REFERENCES**

1. Foster PJ, Buhrmann R, Quigley HA, Johnson GJ. The definition and classification of glaucoma in prevalence surveys. Br J Ophthalmol. 2002;86(2):238-42. Epub 2002/01/30. PubMed PMID: 11815354; PubMed Central PMCID: PMCPMC1771026.

2. Lowe RF. Aetiology of the anatomical basis for primary angle-closure glaucoma. Biometrical comparisons between normal eyes and eyes with primary angle-closure glaucoma. Br J Ophthalmol. 1970;54(3):161-9. PubMed Central PMCID: PMCPMC1207665.

3. Tornquist R. Angle-closure glaucoma in an eye with a plateau type of iris. Acta Ophthalmol (Copenh). 1958;36(3):419-23.

4. Quigley HA. Angle-closure glaucoma-simpler answers to complex mechanisms: LXVI Edward Jackson Memorial Lecture. Am J Ophthalmol. 2009;148(5):657-69 e1. Epub 2009/11/03. doi: 10.1016/j.ajo.2009.08.009. PubMed PMID: 19878757.

5. Tornquist R. Shallow anterior chamber in acute glaucoma; a clinical and genetic study. Acta Ophthalmol Suppl. 1953;39:1-74. PubMed Central PMCID: PMC13050373.

6. Spaeth GL. Gonioscopy: uses old and new. The inheritance of occludable angles. Ophthalmology. 1978;85(3):222-32. Epub 1978/03/01. PubMed PMID: 351502.

7. Etter JR, Affel EL, Rhee DJ. High prevalence of plateau iris configuration in family members of patients with plateau iris syndrome. J Glaucoma. 2006;15(5):394-8. Epub 2006/09/22. doi: 10.1097/01.ijg.0000212253.79831.7a. PubMed PMID: 16988601.

8. Subak-Sharpe I, Low S, Nolan W, Foster PJ. Pharmacological and environmental factors in primary angle-closure glaucoma. Br Med Bull. 2010;93:125-43. Epub 2009/11/26. doi: 10.1093/bmb/ldp042. PubMed PMID: 19933218.
