# Supplementary material for: Mutations in SPATA13/ASEF2 cause primary angle closure glaucoma
Source: PLoS Genet. 2020 Apr 27;16(4):e1008721. doi: 10.1371/journal.pgen.1008721 (PMC7233598; doi:10.1371/journal.pgen.1008721)
Supplement: S3 Text — (DOCX) [file pgen.1008721.s003.docx]

**S3 Text: SNP analysis and clinical details of family 5:II**

One patient, (5:II:2), with PACS carried the same 9 bp *SPATA13* deletion (c.1432_1440del; p.478_480del) identified in Family 1. This patient also came from the East London area but was not known to be related to Family 1. Haplotype analysis using SNPs within *SPATA13* showed that he does not have the same haplotype as Family 1.

| SPATA13-SNP | Family 1-V:15 | Family 1-IV:27 | 5:II:2 |
| --- | --- | --- | --- |
| rs2765163 | AB | AA | BB |
| rs9507223 | AB | BB | AA |
| rs4770571 | AB | BB | AA |
| rs912151 | AB | AA | BB |

He has two daughters (half-siblings), 5:III:1 and 5:III:2 (Figure 2). One, 5.III.2, had PAC, and the other, 5.III.1, was unaffected, neither carried the 9 bp deletion. The mother, 5:II:3, of the affected daughter had narrow but open angles and was hyperopic. The daughter, 5:III:2, with PAC was also hyperopic and suffered severe meningococcal septicaemia requiring intensive care admission at the age of 18 years. She was presumed to be a phenocopy of PACS related to hyperopia, inherited from her mother, possibly amplified by the tendency to positive refractive error in young patients diagnosed with bacterial meningitis [1]. She had shorter biometry than sex-specific means but had a convex iris configuration suggestive of pupil block, rather than plateau iris. No other family members of 5:II:2 were available for this study.

**REFERENCE**

1. Woodruff ME. Differential-Effects of Various Causes of Deafness on the Eyes, Refractive Errors, and Vision of Children. Am J Optom Phys Opt. 1986;63(8):668-75. PubMed PMID: WOS:A1986D614400011.
