# Supplementary material for: Mutations in SPATA13/ASEF2 cause primary angle closure glaucoma
Source: PLoS Genet. 2020 Apr 27;16(4):e1008721. doi: 10.1371/journal.pgen.1008721 (PMC7233598; doi:10.1371/journal.pgen.1008721)
Supplement: S4 Text — (DOCX) [file pgen.1008721.s004.docx]

**S4 Text:** **Pathogenicity of variants**

In total 9 variants were identified in this study. The SIFT score of three of these mutations (p.R89P, p.R363C, p.S473N) are predicted to be deleterious with POLYPHEN score predicting them to be possibly/probably damaging. There are 2 mutations, p.S292I and p.R964L, where SIFT score predicted them to be tolerated and POLYPHEN score predicting them to be probably damaging. Three of the mutations (p.P166L, pS246T and p.A385V) are predicted to be tolerated by SIFT score and benign by POLYPHEN score. Except for one, p.P166L, the Combined Annotation Dependent Depletion (CADD) score of all the variants is above 10 suggesting that except of this variant, the rest are likely to be deleterious. This variant was also identified in our control of European Caucasian cohort.

One of the patients (3:I) harbours a mutation in the RHOGEF domain (c.C2891T, p.P964L) of SPATA13 (Table 1). The proline at 964 is highly conserved in human, chimpanzee, mouse, rat, gray wolf and dog. This patient underwent laser iridotomy, which did not control the IOP. Her PACG was unresponsive to maximal medical therapy and laser iridotomies, requiring trabeculectomy in one eye and lens extraction in the other. She is the only clearly affected subject with significant myopia, but angle closure disease. This patient and patient 9:I with the c.C1154T, p.A385V variant were the only ones that did not present with acutely elevated IOP. Both these patients had significant glaucomatous cupped discs (0.9). Eyes with asymptomatic PACG often present with severe to end-stage visual field (VF) loss at the time of first presentation to hospital[1]. In contrast, most PACG eyes with previous symptomatic angle closure present with mild or moderate VF defects. The visual morbidity of PACG may be related to the finding that the asymptomatic form of the disease is visually destructive.

**REFERENCE**

1. Ang LP, Aung T, Chua WH, Yip LW, Chew PT. Visual field loss from primary angle-closure glaucoma: a comparative study of symptomatic and asymptomatic disease. Ophthalmology. 2004;111(9):1636-40. Epub 2004/09/08. doi: 10.1016/j.ophtha.2004.01.032. PubMed PMID: 15350315.
