# Supplementary material for: Mutations in SPATA13/ASEF2 cause primary angle closure glaucoma
Source: PLoS Genet. 2020 Apr 27;16(4):e1008721. doi: 10.1371/journal.pgen.1008721 (PMC7233598; doi:10.1371/journal.pgen.1008721)
Supplement: S5 Text — (DOCX) [file pgen.1008721.s005.docx]

**S5 Text: Co-localisation analysis of SP-1277 with kinetochore markers, PLK-1 and CENP-E**

Co-localisation was evaluated first using visual methods such as a side-by-side comparison of the two images, with head arrows provided as landmarks and line plots with a line width of 10 pixels by using the ImageJ “RGB line profile” plugin (Laummonerie & Mutterer, Institut de Biologie Moleculaire des Plantes, Strasbourg, France). If there was a clear indication of co-localisation the quantitative methods were applied.

Co-localisation was quantified by performing the automatic thresholding algorithm of Costes and co-workers [1] using ImageJ (rsb.info.nih.gov/ij) plugins “Colocalization test” and “Colocalization Threshold” by T.Collins and W. Rasband. Randomizations were done with 100 iterations (Colocalization Test plugin). If co-localisation was significant (P>0.95) the second plugin “Colocalization Threshold” was applied to determine the co-localisation parameter thresholded Manders Coefficient (tM) as well as the scatterplot which provides a qualitative indication of the degree of co-localisation. The degree of co-localisation of SP-1277 channel with the other channel was expressed as percent of tM value (fraction between 0 and 1). Co-localisation parameters values were confirmed using the image analysis software package Imaris (Bitplane Scientific Software AG) and the JACop plugin for ImageJ.

**REFERENCE**

1. Costes SV, Daelemans D, Cho EH, Dobbin Z, Pavlakis G, Lockett S. Automatic and quantitative measurement of protein-protein colocalization in live cells. Biophys J. 2004;86(6):3993-4003. Epub 2004/06/11. doi: 10.1529/biophysj.103.038422. PubMed PMID: 15189895; PubMed Central PMCID: PMCPMC1304300.
