# Supplementary material for: Mutations in SPATA13/ASEF2 cause primary angle closure glaucoma
Source: PLoS Genet. 2020 Apr 27;16(4):e1008721. doi: 10.1371/journal.pgen.1008721 (PMC7233598; doi:10.1371/journal.pgen.1008721)
Supplement: S6 Text — (DOCX) [file pgen.1008721.s006.docx]

**S6 Text:** **Variable expression and incomplete penetrance in Family 1**

The variable expression was best illustrated by the proband’s two daughters (VI:4 and VI:6, **Fig. 1 and Fig 2A**), both of them carry the 9 bp *SPATA13* deletion. Individual VI:4 was clearly affected and had PIC and PAC, while the other daughter VI:6, was a high myope of -8 dioptres spherical equivalent with pigment in the trabecular meshwork and IOP of 24 mmHg without glaucoma damage and therefore classed as ‘other ocular conditions’.

Similarly, VI:28 and VI:30 also carry the disease allele without manifesting the PAC phenotype, however, both were myopic. PAC is most common in hyperopic eyes, with shorter axial lengths and shallower anterior chambers, resulting in the disease defining feature – iridotrabecular contact (ITC) [1]. ITC is extremely uncommon in myopic eyes with longer axial lengths, which are at increased risk of POAG [2]. The genetics of myopia (largely mediated by axial enlargement of the eye) has been the subject of extensive research and is increasingly well understood through GWAS [3]. In addition to genetic mechanisms underlying development of myopia, it is now thought that refractive errors are caused by illumination-dependent signaling which promotes ocular elongation [3]. It is plausible that the phenotype influenced by mutations in *SPATA13* may be modified by separate processes, which control the axial length of the eye. Our results point strongly towards *SPATA13* influencing risk of angle-closure in small eyes through its effect on the development of the anterior segment of the eye. Our work raises the possibility that *SPATA13* may influence the development of other glaucoma phenotypes, resembling pigment dispersion syndrome in longer, myopic eyes.

The only affected male, V:30, in Family 1 developed PACG at the age of 71 with a mild presentation. His two sons, VI:25 (42y) and VI:26 (48y), and a daughter, VI:27 (43y), carried the 9bp deletion. His sons were classified as ‘uncertain diagnosis’ as they showed presence of trabecular pigment and PIC, so they could develop the disease in the future. The daughter, however, was asymptomatic without any pigmentation and had flat iris configuration. Of the three siblings, she appears less likely to develop PACG and could be an example of a non-penetrant carrier.

Other non-penetrant carriers in Family 1 include two male members VI:36 (24y) and VI:37 (26y) carrying the 9bp deletion. However, as they were too young to manifest the disease phenotype they have been excluded from the calculation of % of penetrance in the family. On the other hand, the two men V:44 (72y) and V:45 (63y) only showed pigmentation in the angle on gonioscopic examination, with no definite angle-closure disease and are therefore considered unaffected. Based on this, it has been calculated that there is a reduced penetrance of approximately 92% in Family 1.

With the limited number of affected males in the family, and the one affected male showing a very mild phenotype, and the majority of non-penetrant carrier being male, it appears that male subjects, have a milder form of the disease, or there may be other protective factors at play. The variable expressivity observed in Family 1 could also indicate that genetic modifiers are leading to the pedigree-specific variable phenotypic expression of the *SPATA13* mutation.

**REFERENCES**

1. Friedman DS, Gazzard G, Foster P, Devereux J, Broman A, Quigley H, et al. Ultrasonographic biomicroscopy, Scheimpflug photography, and novel provocative tests in contralateral eyes of Chinese patients initially seen with acute angle closure. Arch Ophthalmol. 2003;121(5):633-42. Epub 2003/05/14. doi: 10.1001/archopht.121.5.633. PubMed PMID: 12742840.

2. Mitchell P, Hourihan F, Sandbach J, Wang JJ. The relationship between glaucoma and myopia: the Blue Mountains Eye Study. Ophthalmology. 1999;106(10):2010-5. Epub 1999/10/16. doi: 10.1016/s0161-6420(99)90416-5. PubMed PMID: 10519600.

3. Tedja MS, Wojciechowski R, Hysi PG, Eriksson N, Furlotte NA, Verhoeven VJM, et al. Genome-wide association meta-analysis highlights light-induced signaling as a driver for refractive error. Nat Genet. 2018;50(6):834-48. Epub 2018/05/29. doi: 10.1038/s41588-018-0127-7. PubMed PMID: 29808027; PubMed Central PMCID: PMCPMC5980758.
