# Supplementary figures and images for: Mutations in SPATA13/ASEF2 cause primary angle closure glaucoma
Source: PLoS Genet. 2020 Apr 27;16(4):e1008721. doi: 10.1371/journal.pgen.1008721 (PMC7233598; doi:10.1371/journal.pgen.1008721)

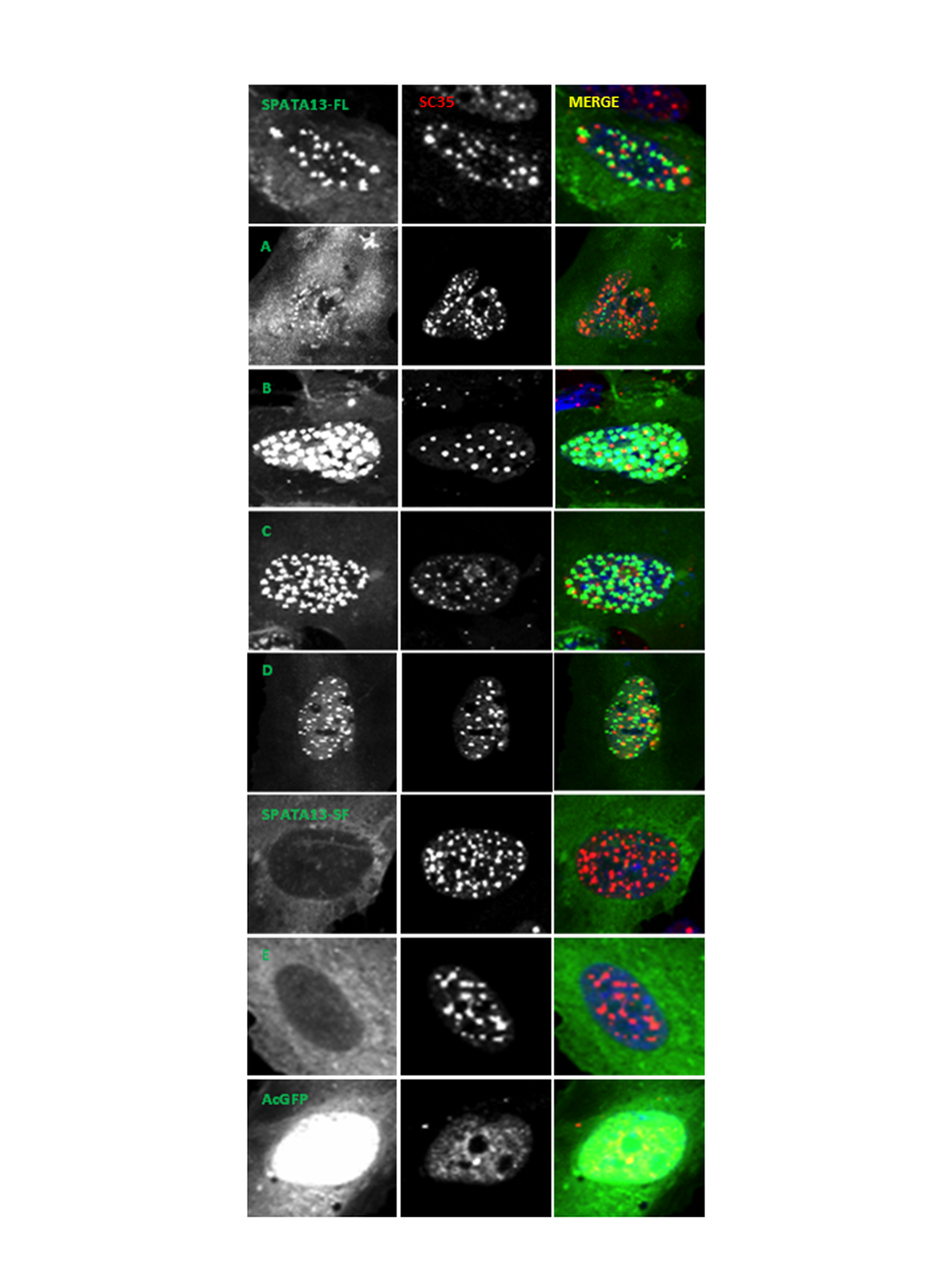

Supplement: S1 Fig — RPE-1 cells were transiently transfected with mutant AcGFP-tagged SPATA13 constructs, and co-stained using anti-SC35 antibody, a marker for nuclear speckles. Mutations are as follows: (A) SP-1277-S292I; (B) SP-1277-S473N; (C) SP-1277-9bp del; (D) SP-1277-P964L; and (E) SP-652-P964L. Mutant proteins did not show co-localisation with SC35. Nuclei were stained using DAPI (blue). (TIF) [file pgen.1008721.s007.tif]
